# Q-nexus: a comprehensive and efficient analysis pipeline designed for ChIP-nexus

**DOI:** 10.1186/s12864-016-3164-6

**Published:** 2016-11-04

**Authors:** Peter Hansen, Jochen Hecht, Jonas Ibn-Salem, Benjamin S. Menkuec, Sebastian Roskosch, Matthias Truss, Peter N. Robinson

**Affiliations:** 1 Institute for Medical and Human Genetics, Charité-Universitätsmedizin Berlin, Augustenburger Platz 1, Berlin, 13353 Germany; 2 Berlin Brandenburg Center for Regenerative Therapies (BCRT), Charité-Universitätsmedizin Berlin, Augustenburger Platz 1, Berlin, 13353 Germany; 3 Centre for Genomic Regulation (CRG), The Barcelona Institute of Science and Technology, Dr. Aiguader 88, Barcelona, 08003 Spain; 4 Universitat Pompeu Fabra (UPF), Barcelona, Spain; 5 Faculty of Biology, Johannes Gutenberg University Mainz, Ackermannweg 4, Mainz, 55128 Germany; 6 Institute of Molecular Biology, Ackermannweg 4, Mainz, 55128 Germany; 7 Institute for Bioinformatics, Department of Mathematics and Computer Science, Freie Universität Berlin, Arnimallee 14, Berlin, 14195 Germany; 8 Labor für Pädiatrische Molekularbiologie, Charité-Universitätsmedizin Berlin, Augustenburger Platz 1, Berlin, 13353 Germany; 9 Max Planck Institute for Molecular Genetics, Inhestr. 63-73, Berlin, 14195 Germany; 10 Current address: The Jackson Laboratory for Genomic Medicine, 10 Discovery Drive, Farmington, 06032 CT USA

**Keywords:** Chromatin immunoprecipitation, ChIP-nexus, ChIP-exo, Duplication rates, Library complexity, Algorithm, Bioinformatics

## Abstract

**Background:**

ChIP-nexus, an extension of the ChIP-exo protocol, can be used to map the borders of protein-bound DNA sequences at nucleotide resolution, requires less input DNA and enables selective PCR duplicate removal using random barcodes. However, the use of random barcodes requires additional preprocessing of the mapping data, which complicates the computational analysis. To date, only a very limited number of software packages are available for the analysis of ChIP-exo data, which have not yet been systematically tested and compared on ChIP-nexus data.

**Results:**

Here, we present a comprehensive software package for ChIP-nexus data that exploits the random barcodes for selective removal of PCR duplicates and for quality control. Furthermore, we developed bespoke methods to estimate the width of the protected region resulting from protein-DNA binding and to infer binding positions from ChIP-nexus data. Finally, we applied our peak calling method as well as the two other methods MACE and MACS2 to the available ChIP-nexus data.

**Conclusions:**

The Q-nexus software is efficient and easy to use. Novel statistics about duplication rates in consideration of random barcodes are calculated. Our method for the estimation of the width of the protected region yields unbiased signatures that are highly reproducible for biological replicates and at the same time very specific for the respective factors analyzed. As judged by the irreproducible discovery rate (IDR), our peak calling algorithm shows a substantially better reproducibility. An implementation of Q-nexus is available at http://charite.github.io/Q/.

**Electronic supplementary material:**

The online version of this article (doi:10.1186/s12864-016-3164-6) contains supplementary material, which is available to authorized users.

## Background

ChIP-seq, which couples chromatin immunoprecipitation with high-throughput sequencing, has enabled researchers to investigate protein-DNA binding on a genome-wide scale [1–3]. ChIP-seq works by cross-linking DNA-protein complexes with formaldehyde followed by fragmentation of the complexes into short stretches of 300–500 base pairs (bp). The fragments are then immunoprecipitated with an antibody specific for the protein of interest, such as a transcription factor (TF) or a modified histone, in order to enrich for DNA fragments bound by the protein prior to next-generation sequencing (NGS). Since the fragment is much longer than the specific protein-DNA binding site (which tend to be on the order of 6–20 bp in length), ChIP-seq “peaks”, representing areas of enrichment for the bound protein, do not directly allow the exact position of protein-DNA binding to be identified.

For this reason, ChIP-exo, an extension of the basic ChIP-seq method, aims to remove DNA segments that surround the binding site of the protein of interest before NGS adapters are attached in order to characterize the exact binding site of proteins more exactly [4]. The protocol for ChIP-exo is similar to the ChIP-seq protocol with the key difference that a 5’-3’ (*λ*) exonuclease is employed to trim the DNA sequences on each strand to within a few bp of the location at which the protein of interest has been cross-linked to the DNA. DNA sequences located 3’ to the cross-linking point remain intact and thus can be used to identify the genomic location of the binding event if they are sufficiently long and located in non-repetitive areas of the genome; on the other hand, non-cross-linked nonspecific DNA is largely eliminated by the exonuclease treatment, which may contribute towards reducing background noise [5, 6].

The ChIP-exo methodology allows protein-DNA binding interactions to be characterized to a level of detail that was not possible with ChIP-seq. The cross-linked DNA-protein complex protects the 5’ ends of bound DNA fragments from exonuclease digestion, with the cleavage occurring about 5–6 bp upstream of the cross-linking point [5], allowing TF binding sites be mapped at high resolution [7–9]. Additionally, the morphology of the ChIP-exo mapped read profiles allows one to discriminate between direct and indirect protein-DNA binding interactions. Profile-based analysis of ChIP-exo signals can uncover structural and functional clues about the interaction and cooperative nature of genomic TF binding [10].

Despite these advantages, the ChIP-exo methodology has a number of shortcomings, including the need for high amounts of input DNA in order to avoid overamplification artifacts resulting from low amounts of starting DNA [11]. Recently, an adaptation of the ChIP-exo procedure was introduced with the goal of addressing these short-comings. ChIP-nexus (chromatin immunoprecipitation experiments with **N**ucleotide resolution through **EX**onuclease, **U**nique barcode and **S**ingle ligation) involves chromatin immunoprecipitation as with standard ChIP-seq, but then proceeds to ligate adapters that contain Illumina-specific sequences, a *Bam*HI site, and a random barcode arranged in such a way that self-circularization can occur following *λ*-exonuclease digestion, which places the random barcode directly adjacent to the “stop” nucleotide resulting from the cross-linked protein-DNA complex. In comparison to the ChIP-exo protocol, ChIP-nexus is more efficient, because for a given fragment only one ligation (instead of two) is needed. Following ligase-mediated circularization, an oligonucleotide with sequence complementary to segment with the *Bam*HI site is added, which enables relinearization of the circles by means of *Bam*HI digestion. Finally, PCR amplification is performed with primers that match the Illumina sequences of the adapter, followed by single-end Illumina sequencing. The random barcode allows multiple reads that correspond to independent molecules but map to the same position to be distinguished from PCR duplicates.

There is currently no software designed specifically for ChIP-nexus, and the computational analysis in the original publication [11] was performed using a number of scripts for preprocessing and MACS2 [12] for peak calling, which was designed for ChIP-seq data and does not take into account the specifics of ChIP-exo and ChIP-nexus data. Although there are software packages specifically developed for ChIP-exo data [13, 14], they do not provide solutions for the extensive preprocessing of the data before peak calling, which comprises quality trimming, adapter clipping and mapping. For ChIP-nexus an additional processing of the mapping has to be performed in order to benefit from the random barcodes. For ChIP-seq the average fragment length is an important parameter for peak calling and downstream analysis and a number of algorithms for estimation have been developed, e.g. the well known cross-correlation method [2, 12]. For ChIP-exo and ChIP-nexus the relevant quantity is the average width of the regions that are occupied by the protein of interest, which is different from the average fragment length. We will refer to such regions as protected regions, because they are protected from 5’-3’ (*λ*) exonuclease digestion. A number of methods have been developed to estimate the size of the protected region and to call peaks in ChIP-exo data [10, 11, 13, 14].

In this work, we present a software package, Q-nexus, for the analysis of ChIP-nexus and ChIP-exo data. Our software implements an all-in-one approach for the preprocessing of ChIP-nexus reads, a novel method for the estimation of the protected-region width and peak calling that can be applied to ChIP-nexus as well as ChIP-exo data. We tested our software on the available ChIP-nexus data and show that our method for the estimation of protected region width provides unbiased signatures that are homogeneous for biological replicates and specific for individual transcription factors. Using the IDR framework [15], we show that our method for ChIP-nexus peak calling outperforms competing methods with respect to the reproducibility of the results. The Q-nexus software as well as an associated tutorial is freely available at https://github.com/charite/Q.

## Results

### Preprocessing and mapping of ChIP-nexus reads

In standard ChIP-seq, multiple reads that map to the same genomic position are usually considered to be duplicates resulting from PCR overamplification during library preparation, and are therefore removed before further analysis. In contrast, in the ChIP-exo and ChIP-nexus protocols, exonuclease digests multiple distinct DNA fragments up to the identical protein-DNA binding site, and therefore reads mapping to the same position are not necessarily PCR duplicated. While ChIP-exo analysis protocols simply do not remove any reads mapping to the same position, ChIP-nexus employs a randomized barcode in the adapter in order to allow PCR duplicated reads (with the same random barcode) to be distinguished from reads originating from distinct molecules (with different random barcodes). We will refer to reads mapping to the same position as identically mapped (IM) reads. Furthermore, we will refer to IM reads with identical barcodes as IMIB reads and to those with unique barcode as IMUB reads. ChIP-nexus assumes that identically mapped reads with identical barcode (IMIB) represent PCR duplicates and only IMUB are utilized for analysis.

**Fig. 1:**
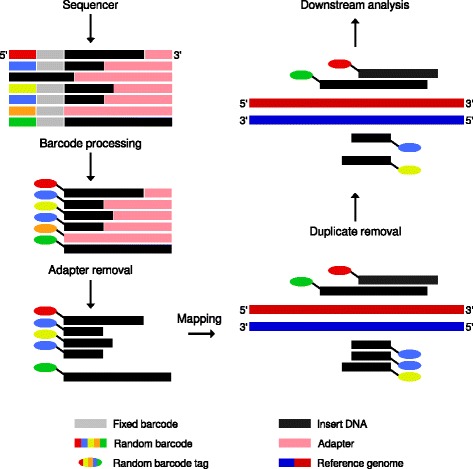
Overview of the Q-nexus preprocessing workflow. During barcode preprocessing, barcode tags are removed. Subsequently, adapter sequences are clipped and reads that consist completely of adapter (*orange-tagged*) are removed. The clipped reads are mapped to the reference genome. The random barcode tags allow PCR duplicated reads and IMUB reads to be distinguished from one another. Only one of the two PCR duplicated reads mapping to the same genomic position (*blue-tagged*) is kept, while reads with different random barcodes are allowed to map to the same position

Existing tools are not able to process these barcodes in the way that is required by the ChIP-nexus protocol. In the original publication, a set of scripts was used to process barcodes and prepare the data for peak calling [11]. We have therefore implemented a preprocessing routine (Fig. 1) that processes raw FASTQ files by extracting the random barcodes from ChIP-nexus reads and writes them into the sequence ID line for downstream analysis. Due to exonuclease digestion a certain proportion of the inserts tend to be very short in size, i.e. shorter than the read length. Therefore, adapter clipping is performed. Following this, alignment of the preprocessed ChIP-nexus reads is carried out with an aligner such as bowtie [16]. The ChIP-nexus protocol assumes that reads that are mapped to the same genomic position and have an identical barcode result from PCR duplication artifacts. For such reads only one read is retained. The Q-nexus software preprocesses a typical ChIP-nexus dataset in less than 17 minutes, where the runtime primarily depends on the number of raw reads to be processed. We compared the results of our preprocessing to those obtained in the original publication [11] and found comparable numbers of IMUB reads (Table 1).

**Table 1:** Preprocessing: IMUB reads and runtime

Factor	Dataset	Reads	IMUB originally	IMUB Q-nexus	Runtime (m)
Dorsal	SRR1175698	18,983,666	6,404,431	6,234,888	6
	SRR1175699	21,926,113	6,219,935	6,054,824	6
Max	SRR1175700	30,781,699	11,100,441	11,381,661	10
	SRR1509031	33,125,449	7,557,857	7,517,583	9
Myc	SRR1175701	27,404,713	3,714,258	4,724,027	6
	SRR1509032	31,044,447	7,366,642	7,480,050	8
Twist	SRR1509029	34,042,864	14,146,225	14,455,901	12
	SRR1509030	92,712,131	30,370,792	30,565,366	31
TBP	SRR1175702	52,656,846	12,924,627	13,349,578	19
	SRR1175703	206,910,978	8,978,714	9,164,510	57

We compared the numbers of IMUB reads of the original analysis [11] and Q-nexus preprocessing. By and large, comparable numbers of reads are mapped. The calculations were performed using four threads on a desktop computer with an Intel Core i7-3770 (3.4GHz). On average, the Q-nexus preprocessing can be performed in less than 17 minutes

### Sequence duplication levels and random barcodes

The complexity of sequencing libraries and PCR overamplification are critical points in virtually all NGS applications [17]. In many NGS applications, a diverse library in which any given sequence appears only once in the final data set is considered ideal, and high levels of duplication generally indicate PCR overamplification or other forms of bias. For this reason, software tools such as FastQC [18] have been developed that generate plots showing the proportion of the overall library with a given degree of duplication, where the sequence duplication level refers to the proportion of reads in the given duplication level bin. ChIP-exo presents a unique challenge to this kind of analysis, because the exonuclease digestion step tends to reduce the overall diversity of starting positions in a library, since distinct starting molecules may be digested down to the same stop position. For this reason, the pile-ups of reads at stop nucleotides cannot be distinguished from PCR duplicated reads. ChIP-nexus was designed to allow PCR duplicates to be identified based on the random barcode [11].

**Fig. 2:**
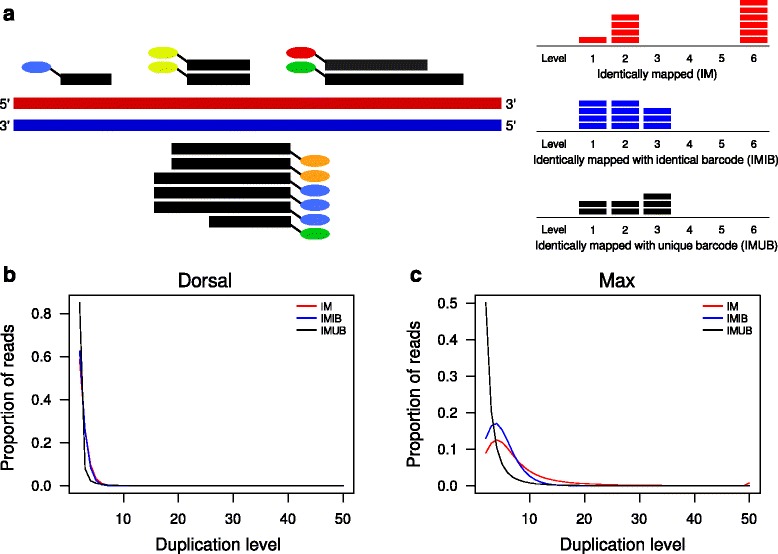
Duplication levels using random barcodes. **a** Toy example of mapped ChIP-nexus reads and the corresponding counts for identically mapped reads (IM, *red*), identically mapped reads with identical random barcodes (IMIB, *blue*), and identically mapped reads with unique random barcodes (IMUB, *black*). The number of horizontal bars for a given level corresponds to the number of reads that have the same level of duplication. Additional file 1: Figure S1 provides a detailed example of how IM, IMIB, and IMUB reads are defined and calculated. **b**, **c** Duplication level plots for a Dorsal dataset with an overall duplication level of 54 % and for a Max dataset with an overall duplication level of 95 %

We developed a bespoke plot that determines the levels of duplication with respect to random barcodes (Fig. 2 a). Instead of considering only the levels of identical sequences or identically mapped reads, we also determine the distribution of the levels for IMIB and IMUB reads. We applied this procedure to all analyzed datasets (Fig. 2 b, c, Additional file 1: Figure S2). Furthermore, we use the various level counts to calculate overall duplication levels for a given sample, defined as the ratio of mapped reads with 5’-end depth > 1 to all mapped reads. For ChIP-nexus, we expect pile-ups of IMUB reads at stop nucleotides, whereas at background positions we expect IMUB reads to occur as singletons. Therefore, the mean per-position depth of IMUB reads can be used to assess the quality of enrichment. We calculated these values for all analyzed datasets (Table 2).

**Table 2:** Duplication levels

Factor	Dataset	IM	IMIB	IMUB
Dorsal	SRR1175698	54 %	50 %	8 %
	SRR1175699	67 %	64 %	8 %
Max	SRR1175700	73 %	65 %	23 %
	SRR1509031	95 %	92 %	33 %
Myc	SRR1175701	85 %	80 %	25 %
	SRR1509032	96 %	92 %	46 %
Twist	SRR1509029	62 %	42 %	35 %
	SRR1509030	82 %	69 %	45 %
TBP	SRR1175702	90 %	87 %	20 %
	SRR1175703	100 %	99 %	30 %

The overall duplication levels for IM, IMIB and IMUB reads were calculated as the proportion of reads with 5’-end depth >1 as a share of all reads (See *Assessment of Q-nexus duplication levels* in Methods)

### Binding characteristics: qfrag length distribution with pseudo-control

**Fig. 3:**
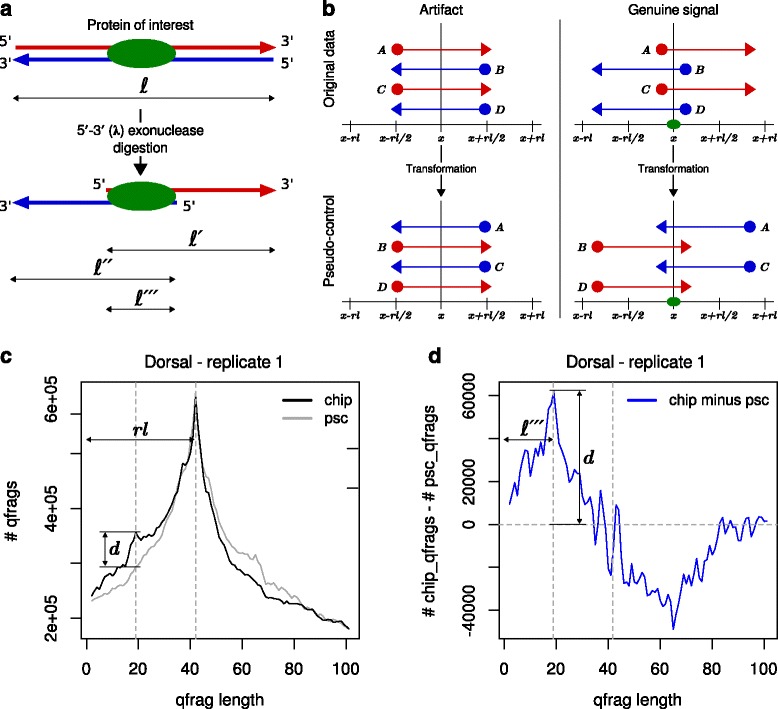
Binding characteristics: qfrag length distribution with pseudo-control. **a** The 5’-3’ (*λ*) exonuclease is employed to trim the DNA sequences of a fragment of length *ℓ* to within a few bp at which the protein of interest has been bound to the DNA. This yields shorter fragments of length *ℓ* ^′^ and *ℓ* ^″^. The width of the protected region (*ℓ* ^‴^) is given by the distance between two 5’ ends on the forward and reverse strand. **b** Schematic representation of the pseudo-control and the corresponding transformation. For mapping artifacts predominantly to be found on chrU and chrUextra (*left-hand*) and for genuine ChIP-nexus peaks (*right-hand*). The pseudo-control is derived from the original mapping data by swapping the strand of each read and subsequently shifting the 5’ end by one read length towards 5’ direction. For artifacts, this has no effect on the qfrag-length distribution in the pseudo-control. **c** qfrag-length distribution for original data (*black*) and pseudo-control (*gray*). Both distributions are are dominated by the phantom peak at one read length. **d** We use the difference between the qfrag-length distributions as signature and the maximum at a length of 19 as estimate for *ℓ* ^′′′^

For ChIP-nexus and ChIP-exo the equivalent of the average fragment length is the width of the regions that are occupied by the protein of interest, which we call “protected-region width”. Such regions are about 6–20 bp, which is much shorter than typically observed average fragment lengths (Fig. 3 a). Here, we present a new method for the estimation of the protected-region width in ChIP-nexus data that uses the distribution of qfrag [19] lengths. A qfrag is defined to be the genomic interval between any pair of 5’ read mapping positions on the forward and reverse strand. We derive the empirical distribution of qfrag-lengths from data by counting the number of qfrags for given lengths. The qfrag-length with the highest number of observed qfrags can be interpreted as the protected-region width (see “Methods”).

It is a well known problem that fragment length estimation by the cross-correlation method [2] can demonstrate an artefactual, “phantom” peak that shows the maximum correlation at a length of one read length [20, 21], that is thought to mainly arise from pile-ups of mapped reads that are arranged in a way that 5’ ends on the forward and reverse strand have a distance of one read length (Additional file 1: Figure S3). Such mapping artifacts are most likely caused by repetitive sequences and for the Drosophila experiments analyzed here (dm3) they occur predominantly on the chromosomes U and Uextra. Similar to the cross-correlation plot, we found that the qfrag length distribution can also be affected by phantom peaks; we therefore developed a method that attempts to remove the phantom peak from the qfrag length distribution in order to enable more accurate estimation of the protected-region width. Our method generates a “pseudo-control” for each ChIP-nexus dataset in which the the strands of each mapped read are swapped and subsequently the 5’ end of each read is shifted by one read length towards 5’ end (see Methods). This transformation has no effect on artifacts responsible for the ’phantom peak’, but it abolishes signals from clusters of qfrags smaller than one read length (Fig. 3 b). Therefore, our procedure subtracts the qfrag counts of the pseudo-control from the counts of the original data and uses the resulting difference as an unbiased signature to estimate the mean protected-region width (Fig. 3 c and d).

### Evaluation: binding characteristics

**Fig. 4:**
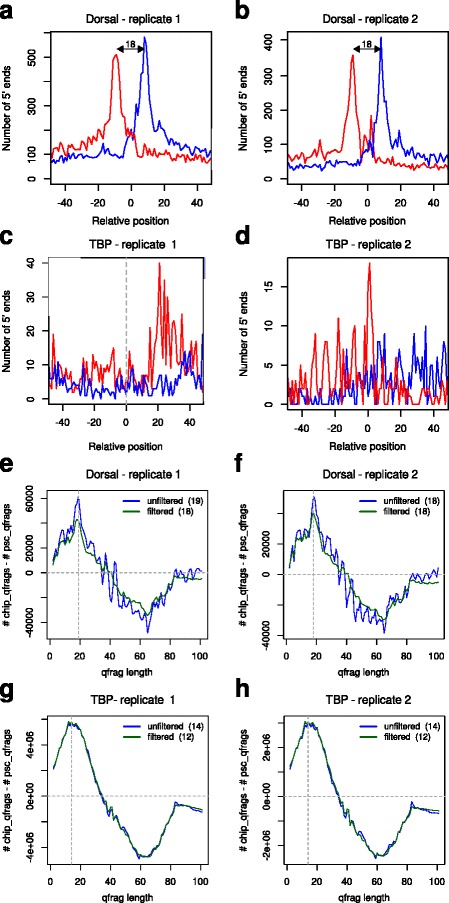
Evaluation: Binding characteristics. **a**, **b** 5’ end coverage around motif centered predicted binding sites for two biological replicates of Dorsal. The two peaks on the forward and reverse strand have a distance of 18 bp, which is in line with a previous analysis [11] of the same data. **c**, **d** Using standardized parameter settings for all samples, no characteristic distribution is derived for TBP. See Additional file 1: Figure S4 for further positive and negative examples. **e**, **f** Difference of qfrag-length distribution between original datasets and pseudo-controls for Dorsal replicates (for all mapped reads (*blue*) and filtered for reads that map to standard chromosomes (green)). The estimated protected-region width of *ℓ* ^′′′^=18 is consistent with the distance derived by the 5’ end coverage around motif centered binding sites. The signatures are independent of whether mapped reads were filtered or not. Furthermore, they are reproducible for biological replicates. **g**, **h** Additional examples for biological replicates of TBP. The signatures are reproducible for biological replicates, but different from the signatures derived for Dorsal. See Additional file 1: Figure S6 for further examples

We applied the method of plotting the 5’ coverage around motif-centered binding sites [10, 11], the cross-correlation method [2], the internal routines of MACS2 [12] and MACE [13] and our Q-nexus method to the ten available ChIP-nexus datasets and compared the estimated distances (Methods and Table 3). The 5’ coverage around binding sites shows maxima on the forward and reverse strand that have distances between 10 and 18 bp (Fig. 4 a, b and Additional file 1: Figure S4) that appear to be reasonable from a biological point of view and are in line with former analysis of the same data [11]. However, we found that the results were unstable and heavily depend on the motif used for selection and centering, as well as the allowed distance between motif and predicted binding site. Using standardized parameter settings the method fails to derive a distinctive distribution in four out of ten cases (Fig. 4 c, d and Additional file 1: Figure S4). The cross-correlation method is obviously strongly biased by the phantom peak and (falsely) estimates in all ten cases the read length of 42 bp (Additional file 1: Figure S5). Also the optimal border pair size estimates between of MACE highly correlate with the read length, indicating a biased estimation. The predicted fragment lengths of MACS2 are indeed smaller than the read length but disagree with the distances that result from the 5’ coverage around binding sites. Our Q-nexus method derives estimates for the protected-region width that are largely consistent with distances derived from the 5’ coverage plots (Fig. 4 a, b and Additional file 1: Figure S4). Finally, the derived signatures (differences between original and pseudo-control) are very similar for biological replicates, but specific for individual factors (Fig. 4 e–h and Additional file 1: Figure S6).

**Table 3:** Evaluation of binding characteristics

Factor	Dataset	5’ end coverage	Cross-correlation	MACS2	MACE	Q-nexus
Dorsal	SRR1175698	18	42	28	47	18
	SRR1175699	18	42	28	62	18
Max	SRR1175700	10	42	19	41	10
	SRR1509031	10	42	32	46	12
Myc	SRR1175701	NA	42	29	42	9
	SRR1509032	NA	42	32	38	12
Twist	SRR1509029	11	42	24	39	12
	SRR1509030	11	42	21	38	12
TBP	SRR1175702	NA	42	37	41	12
	SRR1175703	NA	42	39	42	12

Protected-region width estimates from 5’ coverage around motif centered binding sites, cross-correlation plot, MACS2, MACE (optimal border pair size), and the width of the protected region estimated by Q-nexus. The estimates derived by Q-nexus are consistent with those derived by the method of 5’ coverage around motif centered binding sites

### ChIP-nexus peak calling

**Fig. 5:**
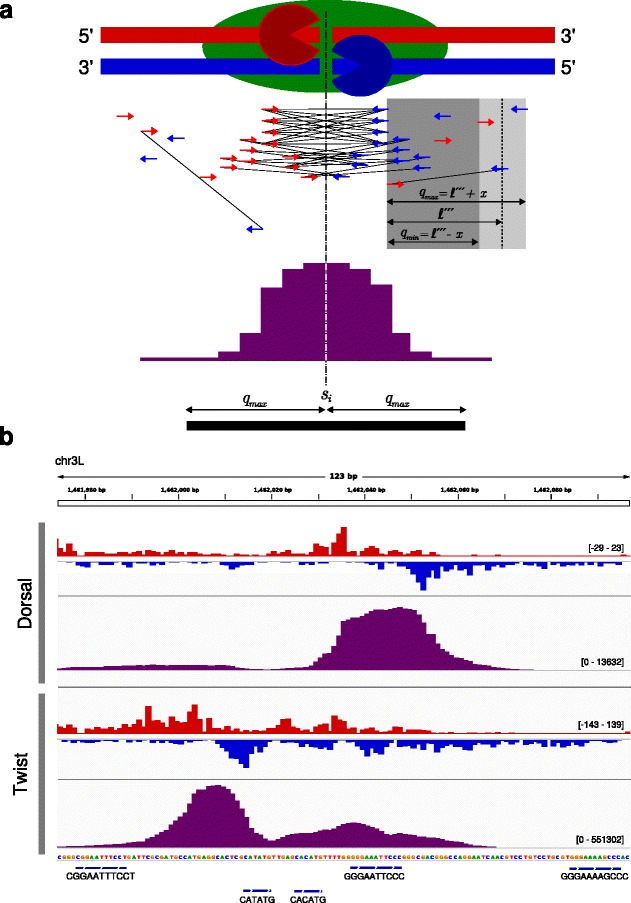
ChIP-nexus peak calling (**a**) Idealized example of a ChIP-nexus peak. The protein of interest (*green*) is bound via one cross-link to the DNA. The 5’ ends are trimmed by exonuclease (’*Pac-Man*’ symbols) up to the cross-link position. 5’ end positions of mapped reads, depicted by *red* and *blue arrows*, are transformed to a qfrags coverage profile (*purple*) along the genome. Local maxima within the qfrag coverage are taken as summits. For each summit position *s*_*i*_ the number of 5’ end positions within a range of *q*_*max*_ is determined and tested for statistical significance. **b** Comparison of 5’ end and qfrags coverage profiles for Dorsal and Twist. 5’ end (*red* and *blue*) and qfrags (purple) coverage profiles at the rho NEE enhancer for Dorsal and Twist (taken from IGV [29]). This region is also shown in the original ChIP-nexus publication [11]. Regions surrounded by clusters of 5’ends on the forward and reverse strand are selectively emphasized by the qfrag method. The qfrags coverage profiles demonstrate two clearly separated peaks for Dorsal and Twist

We implemented an algorithm for ChIP-nexus peak calling which builds on the previous preprocessing steps and accepts mapped reads in BAM format. Since PCR artifacts (IMIB) are already removed, IMUB reads are kept, assuming that such reads stem from different molecules because they have different random barcodes. Our algorithm (see Methods) implements the method of qfrag-length distribution with pseudo-control in order to estimate the protected-region width *ℓ* ^′′′^, which is then used to combine pairs of 5’ ends on the forward and reverse strand by forming qfrags [19] with a minimal allowed distance *q*_*min*_=*ℓ* ^′′′^−5 and a maximal allowed distance of *q*_*max*_=*ℓ* ^′′′^+5. The qfrag-depth at any one position is the total number of qfrags that cover the position. The qfrag coverage has a different depth distribution than the original coverage of reads or 5’ ends. Regions with neighboring clusters of 5’ ends on the forward and reverse strand are selectively emphasized by the qfrag method (Fig. 5). The qfrag coverage profile along the genome is searched for local maxima that we refer to as summits that are then tested for significance. For each summit position the number of 5’ ends that map to within a radius of *q*_*max*_ is determined. *P*-values are calculated using the Poisson distribution and corrected for multiple testing using the Benjamini-Hochberg procedure [22]. The final candidate list is sorted by *P*-value and a cutoff can be specified by the user. Our algorithm does not require fine-tuning of parameters for typical runs.

### Evaluation: reproducibility of peak calling

To evaluate the reproducibility of our peak calling algorithm compared to that of MACS2 and MACE, we used a test framework based on the IDR procedure [15, 23], which has been heavily used to measure the reproducibility of ChIP-seq experiments [20] and should also be applicable to ChIP-nexus data. We performed the comparisons on pairs of biological replicates for five transcription factors (Table 1). We derived peak sets for each dataset using the three peak calling algorithms (see Methods). The peak sets were sorted by significance and the top 100,000 peaks were used for further analysis. The IDR procedure is essentially based on peak overlaps. Two predicted binding positions from two biological replicates were classified as overlapping, if they have a distance of at most 3 bp, which is reasonable given the high resolution provided by the ChIP-nexus protocol.

**Fig. 6:**
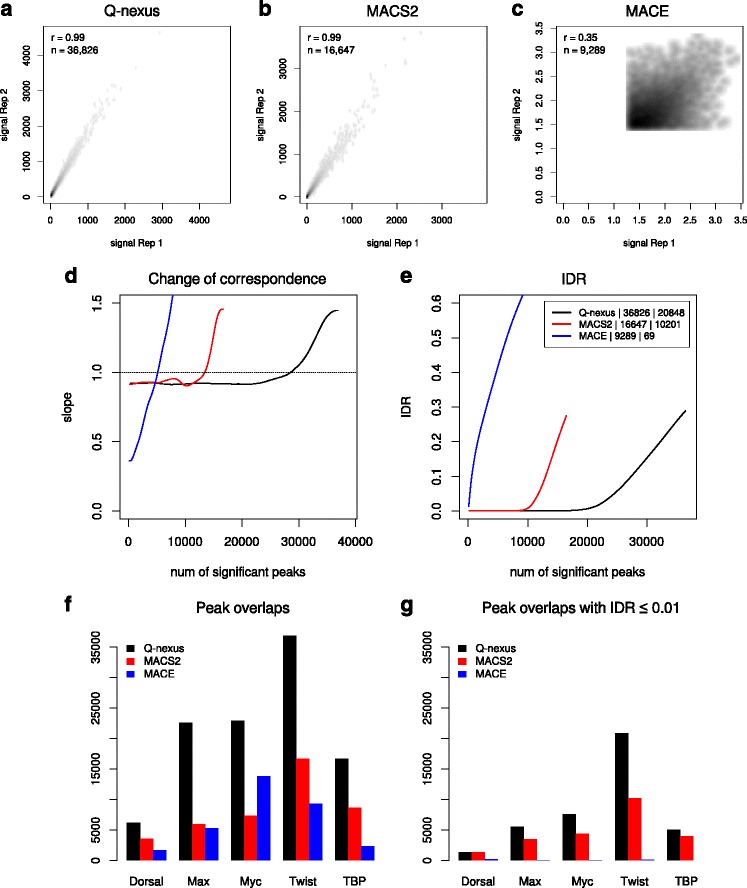
Evaluation: Reproducibility of peak calling. **a-c** We applied the peak calling methods Q-nexus, MACS2, and MACE to a pair of biological replicates of Twist. The scatterplots show the scores of overlapping peaks of the top 100,000 peaks for different methods. The number of overlapping peaks and the Pearson correlation coefficient is given in the upper-left corner of each plot. Q-nexus yields the largest number of overlapping peaks and correlation coefficients of almost 1. **d** The change of correspondence curve shows that peaks derived by Q-nexus remain consistent for 10,000 more than those of MACS2. **e** Q-nexus displays a considerably smaller proportion of irreproducible signals (0.01 < IDR) than MACS2. **f**, **g** We obtained similar results for the other ChIP-nexus datasets of the transcription factors Dorsal, Max, Myc and TBP. For more detailed results see Additional file1: Figures S7 to S16

Figure 6 a–e show the results of the IDR procedure that were obtained for Twist. The top 100,000 peaks derived by Q-nexus display substantial larger overlap compared to MACS2 and MACE (Fig. 6 a–c). It should also be noted that the Pearson correlation coefficients for signal scores of overlapping peaks of MACE are very low in comparison to that of Q-nexus and MACS2. The change of correspondence curve [15], which is used to visualize the transition from reproducible to irreproducible signals, shows that Q-nexus identifies the largest number of reproducible peaks before the transition occurs (Fig. 6 d). Furthermore, according to the IDR, Q-nexus identifies the largest number of reproducible peaks (Fig. 6 e). For all pairs of biological replicates tested similar results as for Twist were obtained (Fig. 6 f–g and Additional file 1: Figures S7–S16 and Tables S1 and S2).

## Discussion

ChIP-nexus is an extension of the ChIP-exo protocol that was shown to outperform ChIP-exo with respect to resolution and specificity, and additionally requires less input material [11]. However, to date no bespoke software for ChIP-nexus analysis has been published, and the original analysis of the ChIP-nexus data was performed using scripts and software such as MACS2 that was originally designed for ChIP-seq [11]. In this work, we present an efficient and easy to use software pipeline for ChIP-nexus data that includes methods for preprocessing and mapping of ChIP-nexus reads, estimation of the protected region width, as well as peak calling. We evaluated our methods on ten publicly available datasets.

One of the major advantages of the ChIP-nexus protocol is the use of random barcodes that allow monitoring of PCR overamplification. Our software recognizes random barcodes and selectively removes PCR duplicated reads while retaining independent reads whose 5’ ends map to same position. Additionally, the random barcode information is used to generate a plot for duplication levels and calculate various statistics that can be used for trouble shooting and optimization.

For ChIP-seq, the size distribution of the fragments needs to be estimated for most peak calling algorithms [4, 24, 25]. For ChIP-nexus, it is not the size of the original fragments that is important, but rather the segment of DNA that cannot be digested because of steric interference by the formaldehyde cross-linked protein (the “protected region”). We present a method for estimating the average width of the protected region that is based on the notion of qfrags [19] and show that, on the ChIP-nexus data, it yields unbiased signatures that are not affected by the so-called phantom peak [20], which is not the case for the cross-correlation method developed for ChIP-seq [2]. The estimates of the protected region width are in line with distances that were derived in a previous study of same data [11] using integrated 5’ end coverage plots around predicted and motif-centered sites. Notably, our method derives signatures that are highly reproducible for biological replicates and specific for different factors.

We have previously developed a method using “qfrag-analysis” to identify candidate peaks in ChIP-seq analysis [19]. Here, we adapted that algorithm for ChIP-nexus analysis. We adopted the peak detection step using the qfrag coverage depth profile along the genome, but for ChIP-nexus data we keep duplicated reads, assuming that they originate from different molecules, and form qfrags using the average protected region width instead of fragment length. Regions bound by the protein of interest are surrounded by pile-ups of 5’ ends reads mapped to the forward and reverse strand and therefore will be emphasized in the qfrag depth profile. This approach differs from previous published peak pairing methods for ChIP-exo [13, 14], in which peaks are detected separately for the forward and reverse strand and subsequently combined into pairs. The saturation-based method we presented for the evaluation ChIP-seq analysis involved a statistical analysis of the number of positions within candidate ChIP-seq peaks to which one or more 5’ read ends mapped. This approach is less suitable for ChIP-exo and ChIP-nexus analysis, in which we expected multiple, independent reads to map to the same position because of the exonuclease digestion. We therefore applied a statistical test based on a standard Poisson model of the count distribution. With respect to the IDR analysis framework applied to biological replicates, our results showed substantially better reproducibility than the other two methods we tested.

## Conclusions

In this study, we present an integrated analysis pipeline implemented in C++ for the analysis of ChIP-nexus and ChIP-exo data. The pipeline begins with efficient methods for preprocessing ChIP-nexus reads to remove PCR duplicates by exploiting information in the random barcodes included in ChIP-nexus adapters to recognize PCR duplicates. This step is skipped for ChIP-exo analysis. We introduce an algorithm that creates pseudo-controls from the data with which true signal can be differentiated from pseudo-peaks, which allows us to accurately estimate the width of the protected region. Our method then performs an analysis of the qfrag distribution to center candidate peaks and then performs a statistical analysis of the read depth distribution to identify peaks. We demonstrate that our method displays a higher reproducibility than other approaches to ChIP-nexus analysis. An efficient and easy-to-use implementation of our method is freely available at https://github.com/charite/Q.

## Methods

### Preprocessing of raw FASTQ reads

We implemented an efficient Q-nexus preprocessing application, flexcat (that is based on flexbar [26]), using the SeqAn C++ programming library [27]. flexcat removes the random and the fixed barcodes, inserts the randomized barcode into the ID field of the sequence (for instance, TL:ATGCC would be added to the description line of a sequence with the random barcode ATGCC), and clips adapter sequences. In ChIP-nexus reads, the random barcode is followed directly by a fixed four-nucleotide barcode. Reads that display no fixed barcode or more than one different nucleotide within the fixed barcode are discarded.

### Read mapping

In principle, Q-nexus can be used with any read mapper, whereby only uniquely mappable reads should be used for downstream analysis. In the experiments described in this work, we used bowtie [16] version 1.1.2 with the settings —chunkmbs 512, -k 1, -m 1, —best, —strata.

### Processing of aligned reads

We implemented an efficient tool called nexcat that scans BAM files in order to identify sets of PCR duplicates. The random barcode from the FASTQ description line is carried over into the BAM file, and can thus be used to distinguish PCR duplicates from IMUB reads. PCR duplicates are identified as sets of reads with an identical random barcode whose 5’ terminus is located at the same genomic position and all but one of those reads are discarded. Since Q-nexus peak calling utilizes only the 5’ end of each read, it does not matter which read is retained.

### Assessment of Q-nexus duplication levels

Our method has three different ways of defining “duplication”. *Identically mapped* (IM) reads, are simply reads whose 5’ end maps to the identical chromosomal location. The information in the random barcodes is not relevant for the determination of IM reads. *Identically mapped with identical barcode* (IMIB) reads are defined as IM reads that additionally have an identical random barcode. The ChIP-nexus analysis pipeline [11] removes all IMIB reads except one at each position. We name the remaining reads *identically mapped with unique barcode* (IMUB) reads (in the original publication, these reads were named “usable reads”). The duplication plots shown in Fig. 2 b–c provide an overview of IM, IMIB, and IMUB reads according to the proportion of reads that have a given duplication level (Fig. 2 a and Additional file 1: Figure S1). We calculate an overall duplication level as the proportion of reads with a duplication level of two or more among all reads. Table 2 shows the overall duplication levels according to each of the three definitions of “duplication”.

### Binding characteristics: qfrag length distribution and pseudo control

We refer to 5’ end positions of reads that map either to the forward or the reverse strand as hits. The outcome of ChIP-nexus experiment is modeled as a set of hits:

$${} T=\{\ h=(\text{pos},\text{strand})\ |\ \text{pos} \in \{1,\ldots,l\} \wedge \text{strand} \in \{f,r\}\},  $$

where *l* is the length of the chromosome. A qfrag is defined to be the genomic interval between an ordered pair of hits (*h*_*i*_,*h*_*j*_), such that *h*_*i*_ is on the forward strand, *h*_*j*_ is on the reverse strand. For the distribution of qfrag-lengths qfrags of fixed lengths are considered and for each length *δ*=2,…,*Δ* the number of qfrags is determined:

$$Q_{t}(\delta)=|\{(h_{i},h_{j})\ |\ h_{i} \in T_{f}\ \land\ h_{j} \in T_{r}\ \land h_{j}-h_{i}=\delta\}|. $$

The pseudo-control is derived from the original data by inverting the strand information for each given hit, i.e

$$\begin{array}{@{}rcl@{}} h^{\prime}.strand:=\left\{ \begin{array}{ll} f,& \text{if}\ h.strand=r\\ r, & \text{otherwise} \end{array}\right. \end{array} $$

and subsequently shifting the (strand inverted) hit by one read length *rl* towards 5’ end, i.e.

$$\begin{array}{@{}rcl@{}} h^{\prime}.pos:=\left\{ \begin{array}{ll} h.pos+rl-1,& \text{if}\ h^{\prime}.strand=r\\ h.pos-rl+1, & \text{otherwise} \end{array}\right. \end{array} $$

The distribution of qfrag-length in the pseudo-control is defined as before:

$$Q_{p}(\delta)=|\{(h^{\prime}_{i},h^{\prime}_{j})\ |\ h^{\prime}_{i} \in T^{\prime}_{f}\ \land\ h^{\prime}_{j} \in T^{\prime}_{r}\ \land h^{\prime}_{j}-h^{\prime}_{i}=\delta\}|. $$

We use the difference between *Q*_*t*_(*δ*) and *Q*_*p*_(*δ*) as signature and the maximum value as estimate for the protected-region width *ℓ* ^′′′^, i.e.

$$\ell^{\prime\prime\prime}={\underset{\delta}{\text{arg max}}}\, Q_{t}(\delta)-Q_{p}(\delta). $$

### Evaluation: Binding characteristics

#### 5’ end coverage around motif centered binding sites

For each dataset summits were derived using Q [19] with the following parameter settings: —fragment- length-average 15, —fragment-length- deviation 10, —keep-dup and sorted by significance. The top 2,000 peaks (summit position ± 40) were used for a de novo motif analysis with DREME [28] using default settings. The top 30,000 peaks were filtered for those with an occurrence of the top motif in a distance of at most the length of the motif. Finally, the selected summits were centered to the center of the motif occurrence. Around the motif filtered and centered sites the integrated distribution of 5’ ends were determined using Q with the following parameter settings: —bed-hit-dist <CENTERED_SITES_BED>,—keep-dup, —pseudo-control.

#### Cross-correlation analysis

We performed cross-correlation analysis using the function get.binding.characteristics of the SPP package (version 1.11) with the following parameter settings: srange=c(2,110),bin=1.

#### MACS2

The predicted fragment lengths were derived in the course of peak calling with parameter settings as stated below.

#### MACE

The optimal border pair sizes of MACE were derived in the course of peak calling with parameter settings as stated below.

#### qfrag-length distribution with pseudo-control

We derived qfrag-length distributions using Q with the following parameter settings: —qfrag-length-distribution, —step-num 110, —keep-dup.

### ChIP-nexus peak calling

We use *q*_*min*_=*ℓ* ^′′′^−*x* and *q*_*max*_=*ℓ* ^′′′^+*x* to form qfrags from all hits on the forward and reverse strand that satisfy *q*_*min*_≤*hj*.*pos*−*hi*.*pos*≤*q*_*max*_. We used a value of *x*=5 by default, which is a parameter for the deviation from estimated protected-region width.

We calculate the depth of qfrags at any given position and search the qfrag coverage profile along the genome for free-standing local maxima which we refer to as summits that correspond to predicted binding positions, where free-standing means that there is no position with a higher qfrag depth within a radius of *q*_*min*_. For each summit *s*_*i*_ the number of 5’ ends within the range *s*_*i*_−*q*_*max*_,…,*s*_*i*_+*q*_*max*_, denoted as *k*, is determined. Assuming a null model in which reads are evenly distributed across the genome, *P*-values are calculated using the Poisson distribution.

$$P(x \geq k)=1-\sum_{i=0}^{k-1}\text{Pois}(i,\lambda) $$

where

$$\lambda=2 \cdot q_{max} \cdot \frac{|T_{f}|+|T_{r}|}{l} $$

All regions covered by at least one qfrag are tested. P-values are corrected for multiple testing using the Benjamini-Hochberg procedure [22].

### Evaluation: reproducibility of peak calling

IDR analyses were performed with parameter settings recommended for pairs of biological replicates. Peak lists were derived for Q-nexus, MACS2 and MACE as stated below.

#### Peak calling parameters for Q-nexus

We used version 1.3.0 of Q with the following parameter settings: —nexus-mode, —top-n 200,000. Q-nexus predicts single binding positions or summits. The summits were extended by 2 bp in upstream and downstream direction, sorted by signal score, i.e. the number of 5’ ends that map for a given summit *s*_*i*_ into the range *s*_*i*_−*q*_*max*_,…,*s*_*i*_−*q*_*max*_, and only the top 100,000 were kept.

#### Peak calling parameters for MACS2

We used version 2.1.0.20150731 of MACS2 in the callpeak mode with the following parameter settings —keep-dup all, —pvalue 5e-01, —call- summits. Furthermore, we used —gsize dm for Drosophila and —gsize hs for Human to specify the size of the genome. MACS2 tends to combine multiple adjacent summits into broader broader peaks and only reports the highest summit position, but the option —call-summits causes MACS2 to report all summits. The summits were extended by 2 bp in upstream and downstream direction, sorted by P-value, and only the top 100,000 were kept.

#### Peak calling parameters for MACE

We used version 1.2 of MACE. The python script for preprocessing was used with the following parameter settings: —kmerSize 0, which turns off the nucleotide bias correction. We did this, because according to the implementation the length of each read has to be greater than three times the kmer-size. Discarding all (clipped) reads shorter than 19 leads to a significant loss of information. The python script for peak calling was used with the following parameter settings: —pvalue 0.99. The MACE algorithm does not report peaks, but border pairs, i.e. pairs of peaks on the forward and reverse strand with a distance that approximates to a optimal border pair size that is estimated from the data. We defined the centers between the border pairs as summits. The summits were extended by 2 bp in upstream and downstream direction, sorted by *P*-value, and only the top 100,000 were kept.

## Additional file

Additional file 1
**Supplementary figures and tables.** The following additional data are available with the online version of this paper. Additional data file 1 contains an explanatory figure for duplication levels as well as figures and tables for additional analyses including duplication rate plots, examples for mapping artifacts, 5’ end coverage around motif centered binding sites, cross-correlation plots, qfrag-length distributions, scatterplots of signal scores of overlapping peaks and corresponding IDR plots, as well as two tables containing the total numbers of overlapping peaks and overlapping peaks with IDR ≤ 0.01 for all pairs of biological replicates. (PDF 3840 kb)

## Abbreviations

Bp: Base pairs
IM: Identically mapped
IMIB: Identically mapped with identical barcode
IMUB: Identically mapped with unique barcode
IDR: Irreproducible discovery rate
