# Supplementary material for: Q-nexus: a comprehensive and efficient analysis pipeline designed for ChIP-nexus
Source: BMC Genomics. 2016 Nov 4;17:873. doi: 10.1186/s12864-016-3164-6 (PMC5097360; doi:10.1186/s12864-016-3164-6)
Supplement: Additional file 1 — Supplementary figures and tables. The following additional data are available with the online version of this paper. Additional data file 1 contains an explanatory figure for duplication levels as well as figures and tables for additional analyses including duplication rate plots, examples for mapping artifacts, 5’ end coverage around motif centered binding sites, cross-correlation plots, qfrag-length distributions, scatterplots of signal scores of overlapping peaks and corresponding IDR plots, as well as two tables containing the total numbers of overlapping peaks and overlapping peaks with IDR ≤ 0.01 for all pairs of biological replicates. (PDF 3840 kb) [file 12864_2016_3164_MOESM1_ESM.pdf]

Q-nexus: A comprehensive and efficient analysis pipeline designed for ChIP-nexus data

- Supplementary Material -

Peter Hansen[1,2], Jochen Hecht[3,4], Jonas Ibn-Salem[5,6], Benjamin S. Menküc[1], Sebastian Roskosch[7], Matthias Truss[8], Peter N. Robinson[1,2,7,9,10]

[1]*Institute for Medical and Human Genetics, Charité-Universitätsmedizin Berlin, Augustenburger Platz 1, 13353 Berlin, Germany.*

[2]*Berlin Brandenburg Center for Regenerative Therapies (BCRT), Charité-Universitätsmedizin Berlin, Augustenburger Platz 1, 13353 Berlin, Germany.*

[3]*Centre for Genomic Regulation (CRG), The Barcelona Institute of Science and Technology, Dr. Aiguader 88, 08003 Barcelona, Spain*

[4]*Universitat Pompeu Fabra (UPF), Barcelona, Spain*

[5]*Institute of Molecular Biology, Ackermannweg 4, 55128 Mainz, Germany*

[6]*Faculty of Biology, Johannes Gutenberg University Mainz, Ackermannweg 4, 55128 Mainz, Germany*

[7]*Institute for Bioinformatics, Department of Mathematics and Computer Science, Freie Universität Berlin, Takustrasse 9, 14195 Berlin, Germany.*

[8]*Labor für Pädiatrische Molekularbiologie, Charité-Universitätsmedizin Berlin, Augustenburger Platz 1, 13353 Berlin, Germany.*

[9]*Max Planck Institute for Molecular Genetics, Inhestr. 63-73, 14195 Berlin, Germany.*

[10]*Current address: The Jackson Laboratory for Genomic Medicine, 10 Discovery Drive, Farmington, CT 06032, United States.*

Contents

Figure S1 Duplication levels - Illustration 3
Figure S2 Duplication level plots 4
Figure S3 IGV screenshots of mapping artifacts 5
Figure S4 5' end coverage around motif centered binding sites 6
Figure S5 Binding characteristics - Cross-correlation 7
Figure S6 qfrag-length distribution with pseudo-control 8
Figure S7 Scatterplots for overlapping peaks - Dorsal 9
Figure S8 Scatterplots for overlapping peaks - Max 10
Figure S9 Scatterplots for overlapping peaks - Myc 11
Figure S10 Scatterplots for overlapping peaks - Twist 12
Figure S11 Scatterplots for overlapping peaks - TBP 13
Figure S12 Correspondence and IDR - Dorsal 14
Figure S13 Correspondence and IDR - Max 15

Figure S14 Correspondence and IDR - Myc 16
Figure S15 Correspondence and IDR - Twist 17
Figure S16 Correspondence and IDR - TBP 18
Table S1 Peak overlaps 19
Table S2 Peak overlaps with IDR ≤ 0.01 19

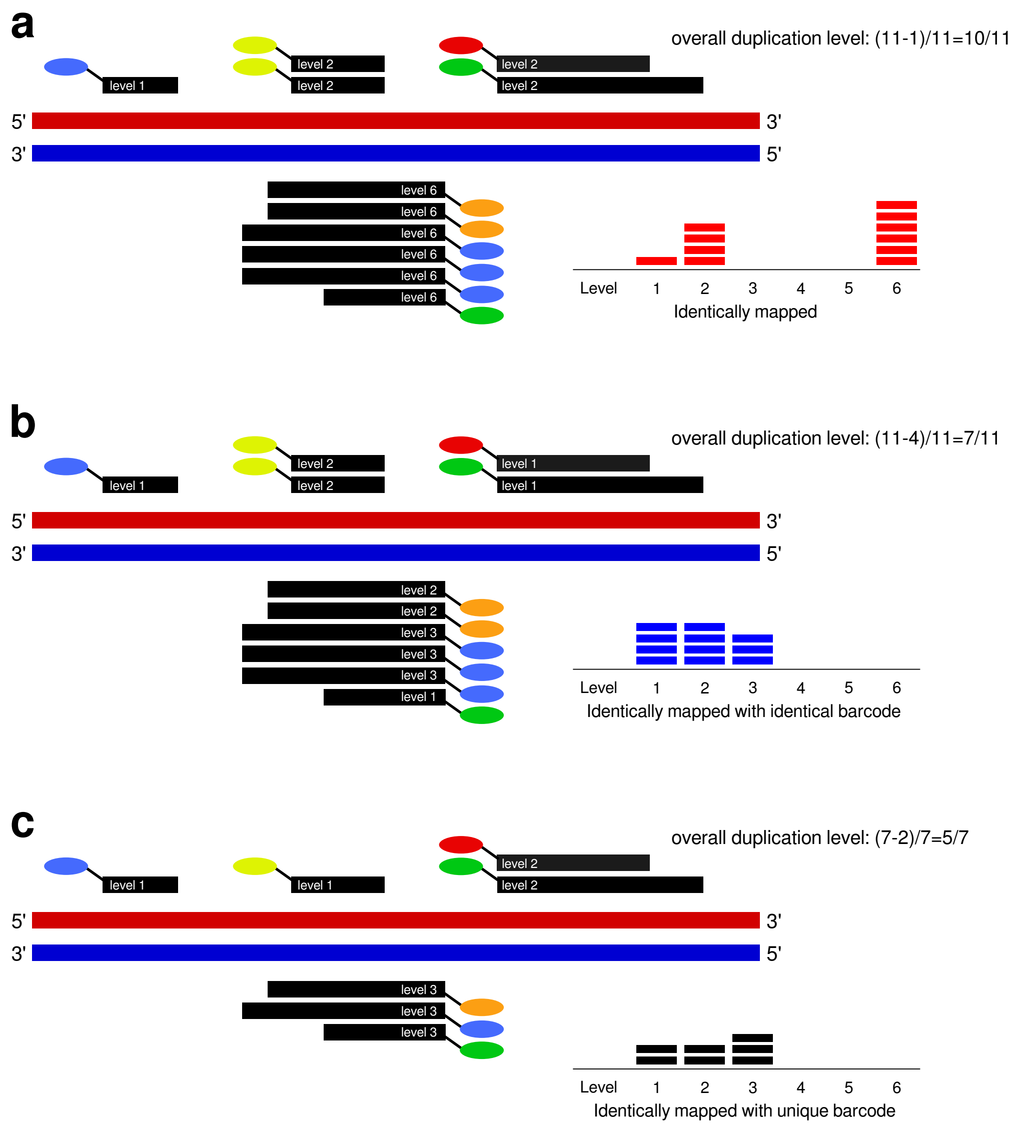

Figure S1. Duplication levels - Illustration. The number of horizontal bars for a given level corresponds to the number of reads that have the same level of duplication. Our method has three different ways of defining "duplication". In panels **(a)-(c)**, we show how 11 hypothetical reads that have been mapped to a chromosome are processed. In panel **(a)**, identically mapped (IM) reads are shown. IM reads are defined to be any reads whose 5' ends map to the same chromosomal location. The information in the random barcodes is not relevant for the determination of IM reads. We see there is only one read that is mapped to a position to which no other read is mapped, hence there is one read for level 1; four reads have a level of 2; and 6 reads have a level of 6. In panel **(b)**, identically mapped with identical bar code (IMIB) reads are analyzed. The total read count is the same as for the IM reads, but the distribution of reads over the various levels can be different for IM and IMIB reads. In this example, there are four reads with a level of one, four reads with a level of 2, and three reads with a level of 3. **(c)** For ChIP-nexus, at each position all but one identically mapped read with identical random barcode are removed (black bars). The remaining reads are called identically mapped, unique barcode (IMUB) reads. In this example, there are two reads with a level of one, two reads with a level of 2, and three reads with a level of 3. We define the overall duplication level for IM, IMIB and IMUB reads as the ratio of the number of reads with a level greater than 1 to the total number of reads.

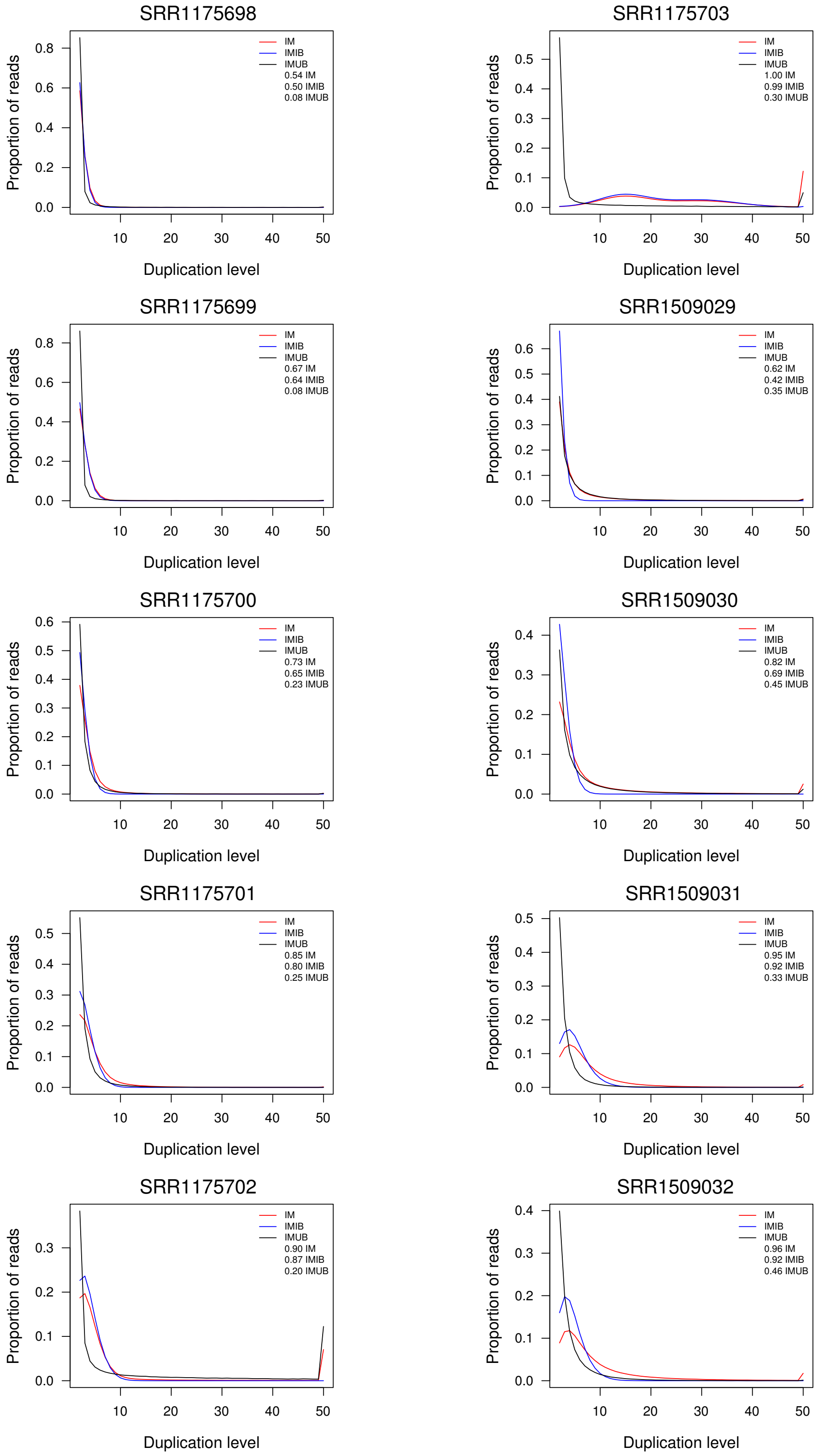

Figure S2. Duplication level plots. Duplication levels for identically mapped reads (IM; red), identically mapped reads with identical barcode (IMIB; blue), and identically mapped reads with unique barcode (IMUB; black). A more detailed explanation of the different duplication levels is presented in the main manuscript and in Figure S1.

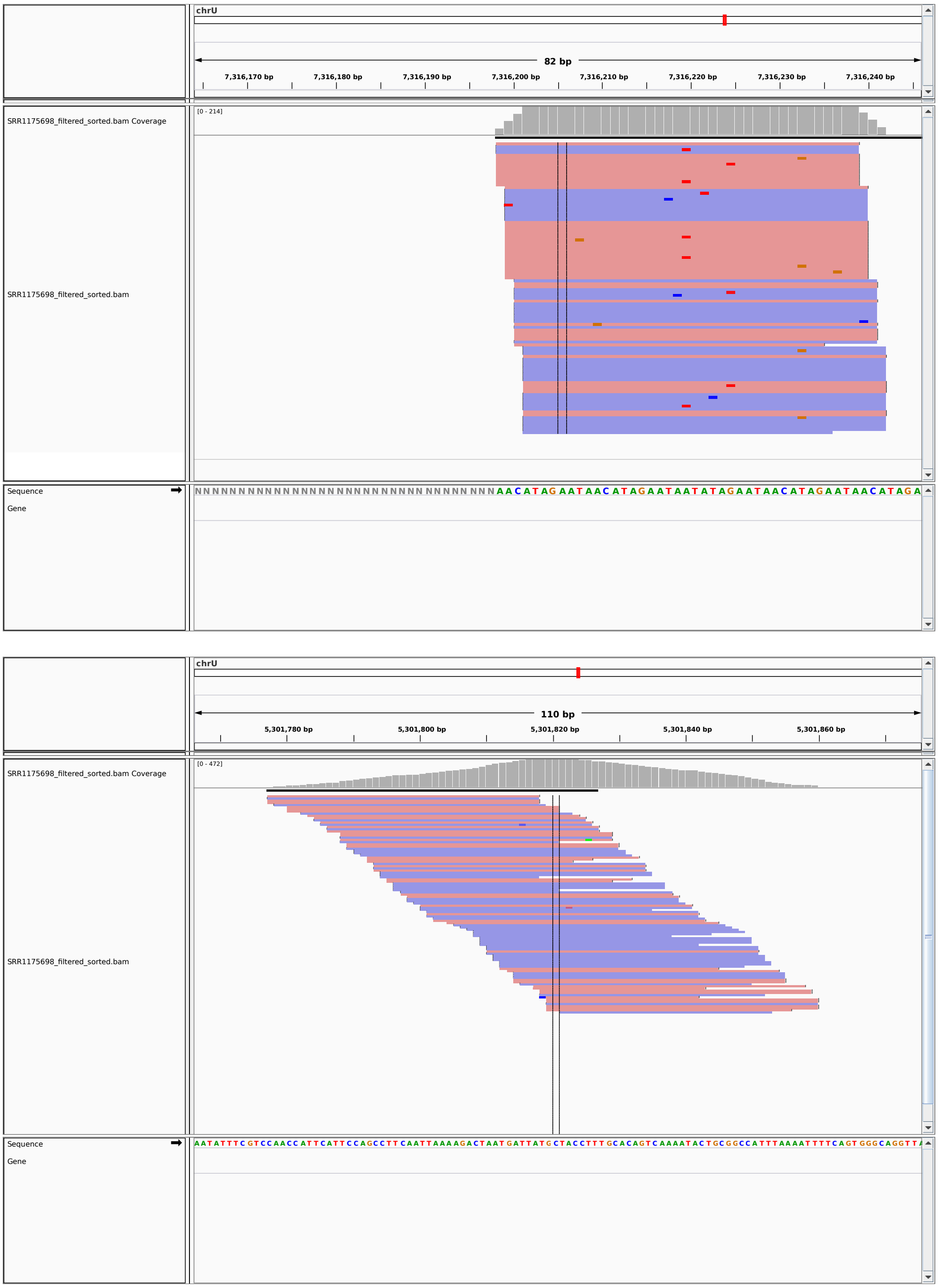

Figure S3. IGV screenshots of mapping artifacts. Two typical mapping artifacts from the Dorsal dataset on chrU. Such artifacts contribute to the phantom peak in the cross-correlation plot and qfrag length distribution.

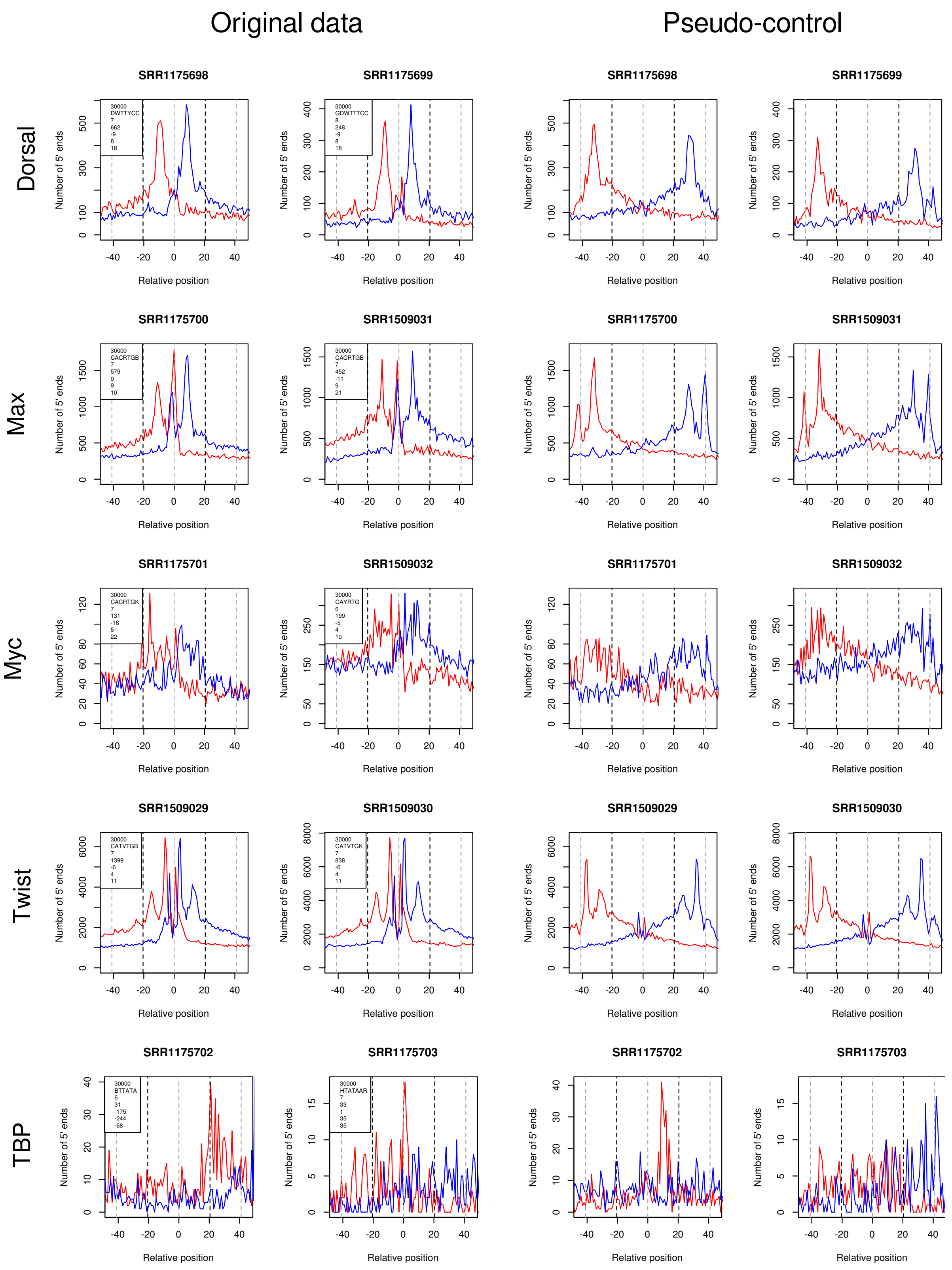

Figure S4. 5' end coverage around motif centered binding sites. First two columns: Integrated counts of 5' ends around motif centered binding sites (Methods). Third and fourth column: Integrated counts of 5' ends around motif centered binding sites after pseudo-control transformation. The Methods section of the main manuscript provides detailed information about how the plots were generated.

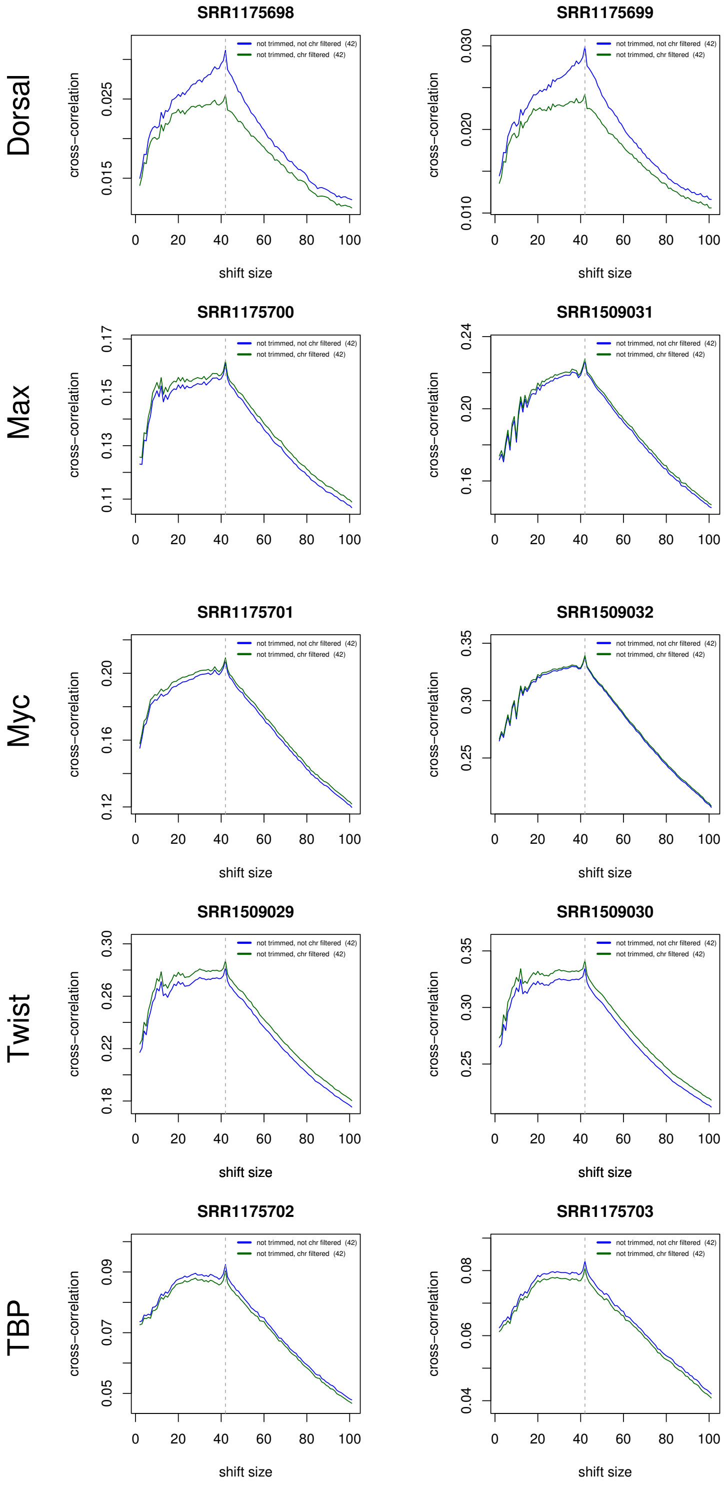

Figure S5. Binding characteristics - Cross-correlation Binding characteristics were derived by applying the conventional cross-correlation method (Kharchenko 2008) to different sets of mapped reads - all mapped reads (blue) and the subset of reads mapped to chr2L, chr2R, chr3L, chr3R, chr4 or chrX (green).

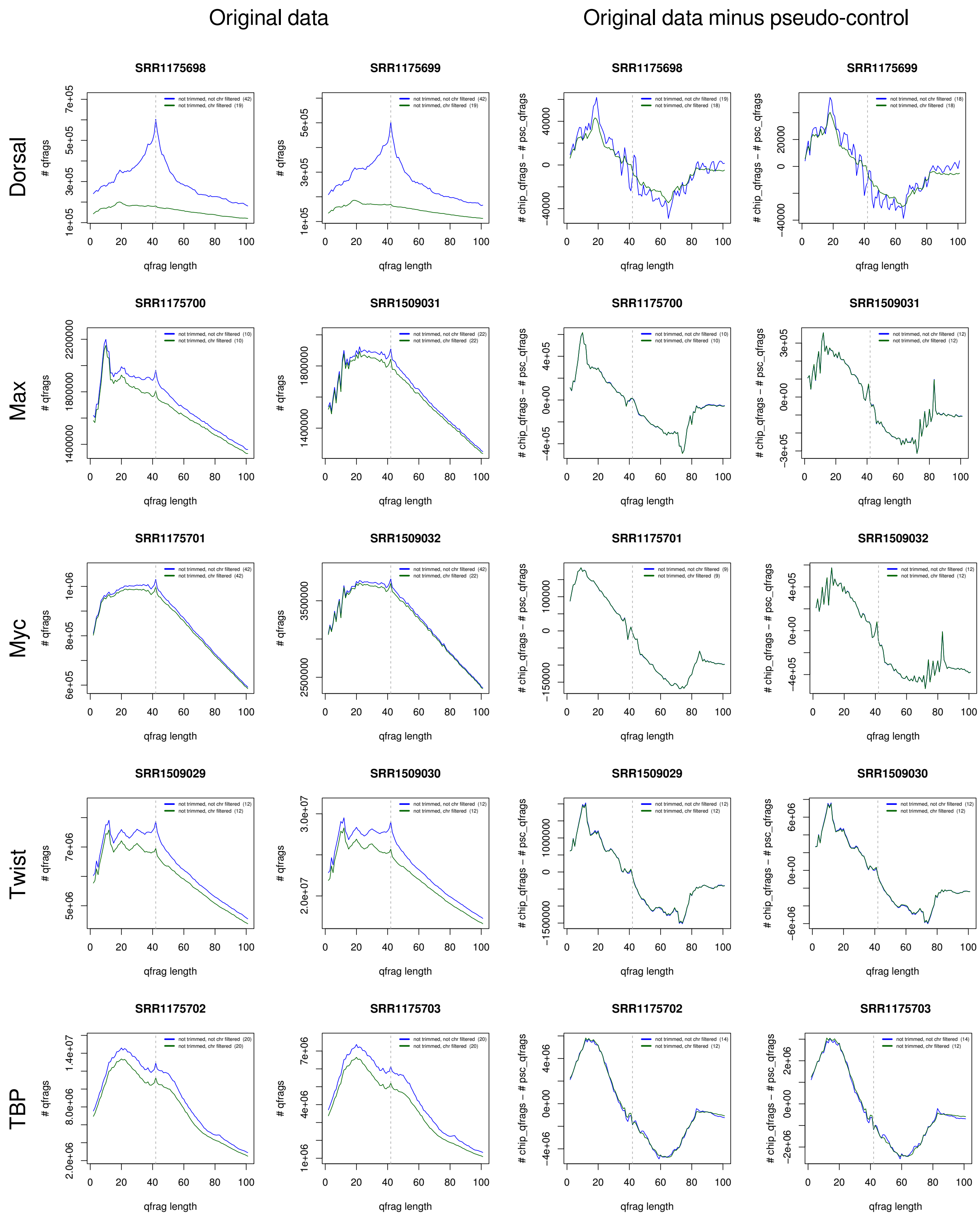

Figure S6. qfrag-length distribution with pseudo-control. qfrag-length distributions were derived for all mapped reads (blue) and only reads mapping to chr2L, chr2R, chr3L, chr3R, chr4 or chrX (green). The distribution of qfrag-lengths is shown for two biological replicates before (columns 1 and 2) and after subtraction of the pseudo-control (columns 3 and 4).

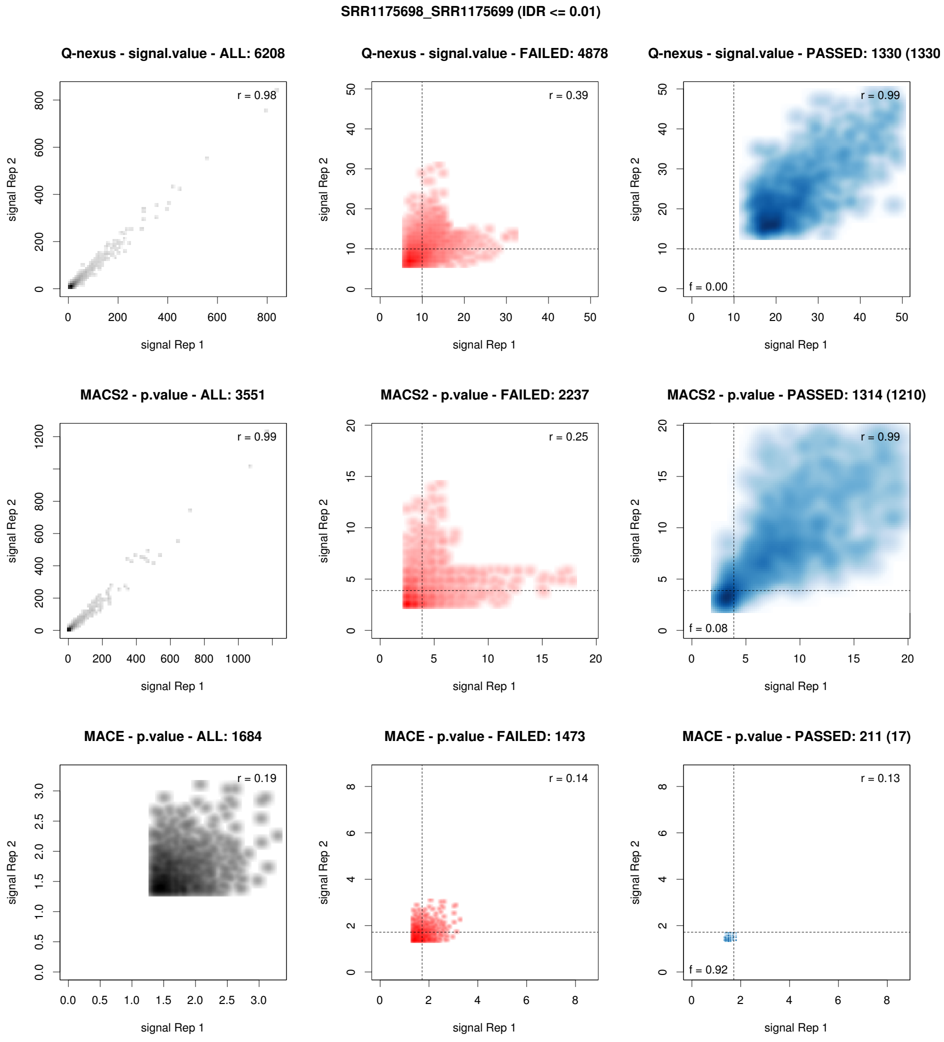

Figure S7. Scatterplots for overlapping peaks - Dorsal. Scatterplots of the $-\log(p)$ P-values for pairs of biological replicates for Q-nexus (first row), MACS2 (second row) and MACE (third row). The plots in the left column show all pairs of $-\log(p)$ P-values (black), and the plots in the middle and left column are zoomed in and show pairs of $-\log(p)$ P-values with IDR > 0.01 (red) and IDR $<= 0.01$ (blue).

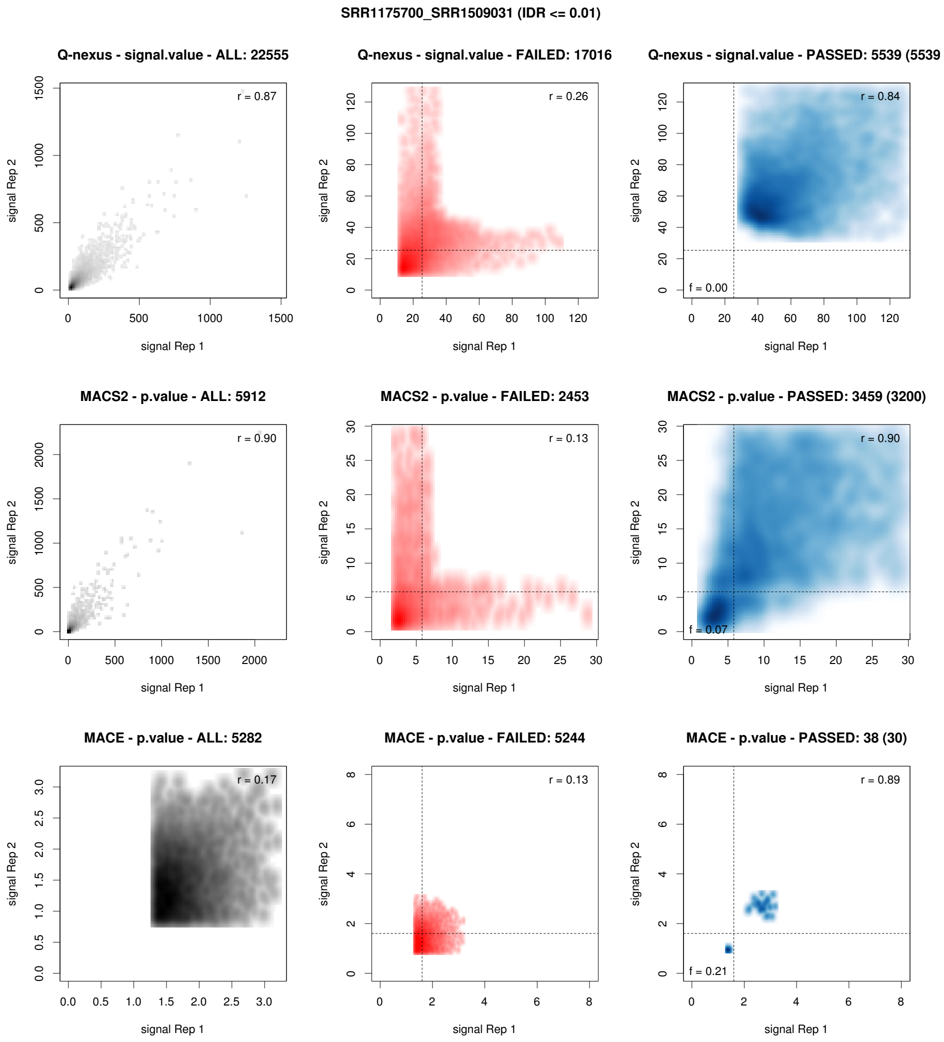

Figure S8. Scatterplots for overlapping peaks - Max. See the legend to Figure S7 for explanations.

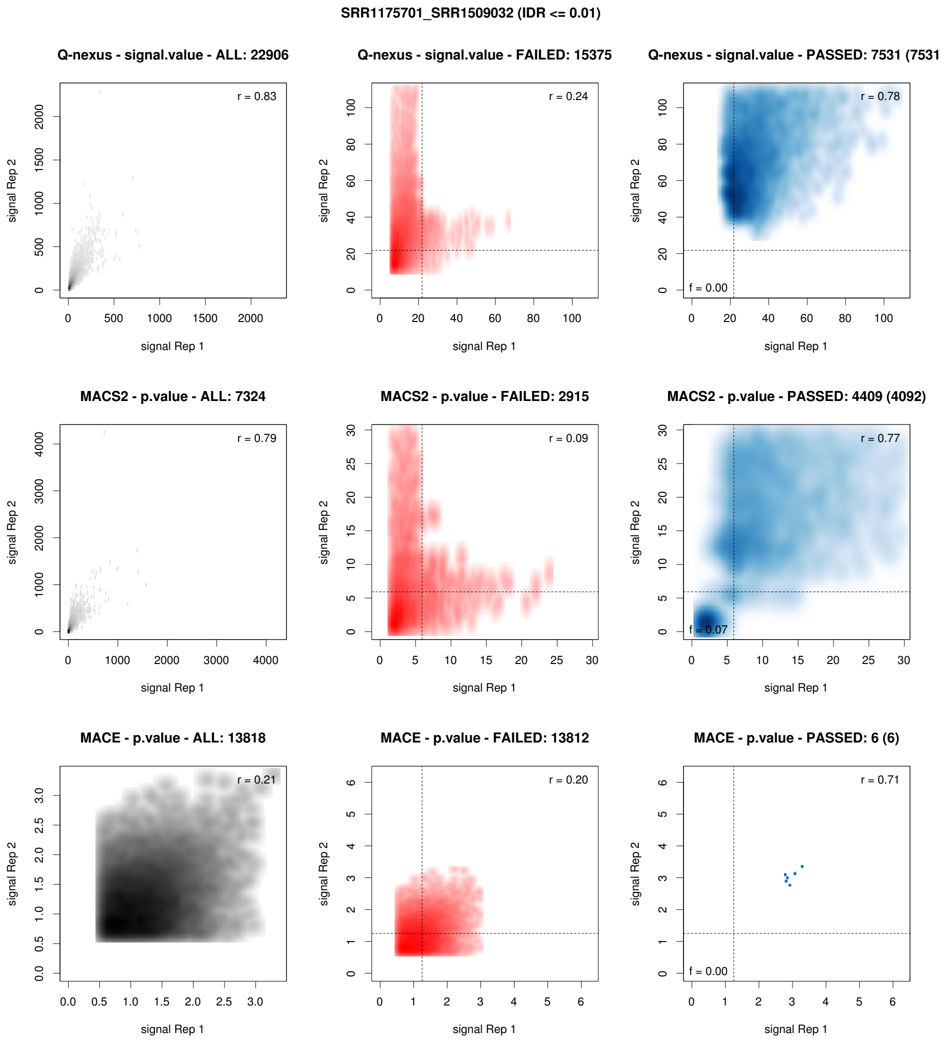

Figure S9. Scatterplots for overlapping peaks - Myc. See the legend to Figure S7 for explanations.

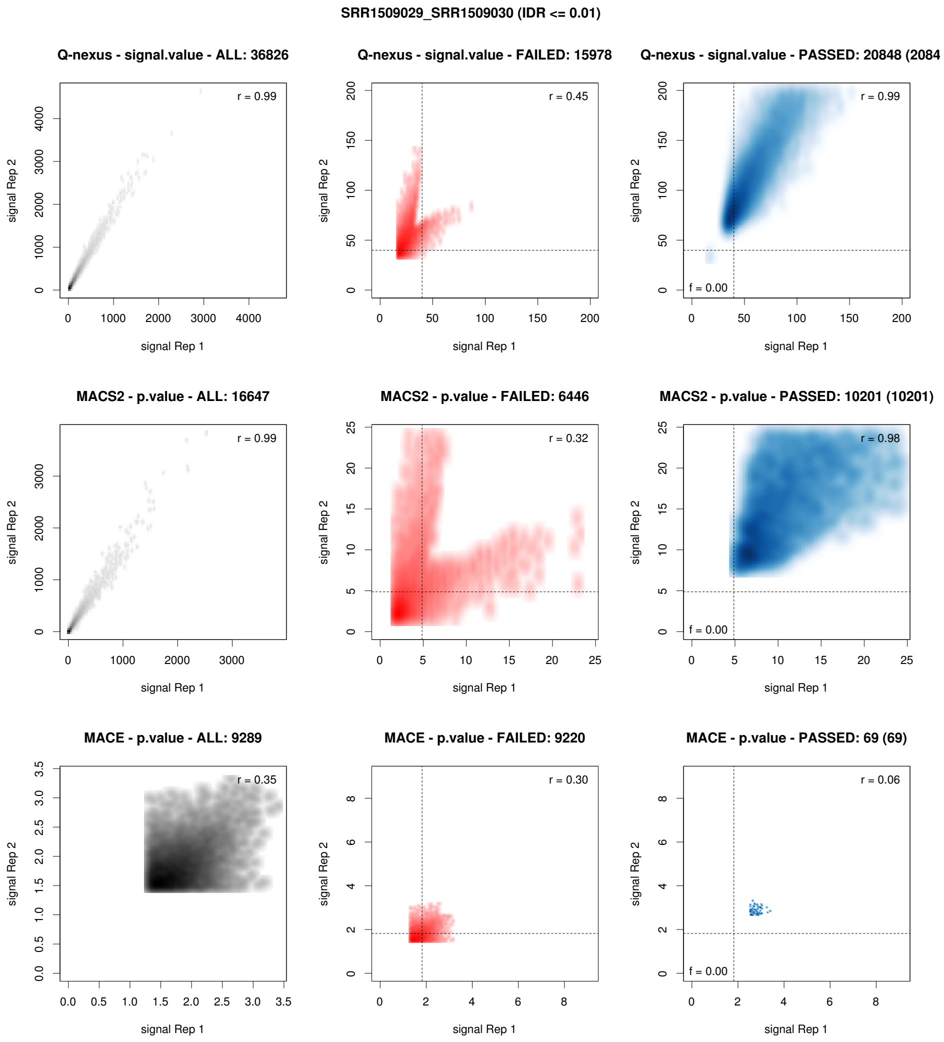

Figure S10. Scatterplots for overlapping peaks - Twist. See the legend to Figure S7 for explanations.

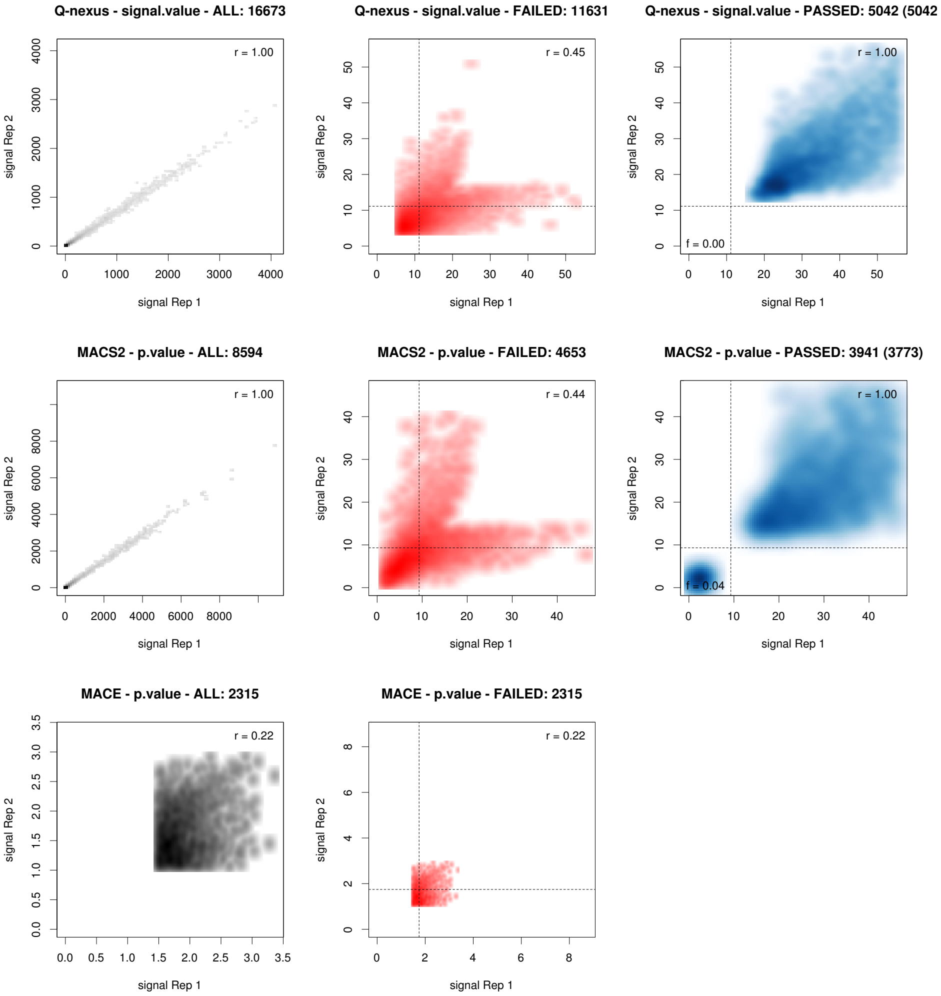

Figure S11. Scatterplots for overlapping peaks - TBP. See the legend to Figure S7 for explanations.

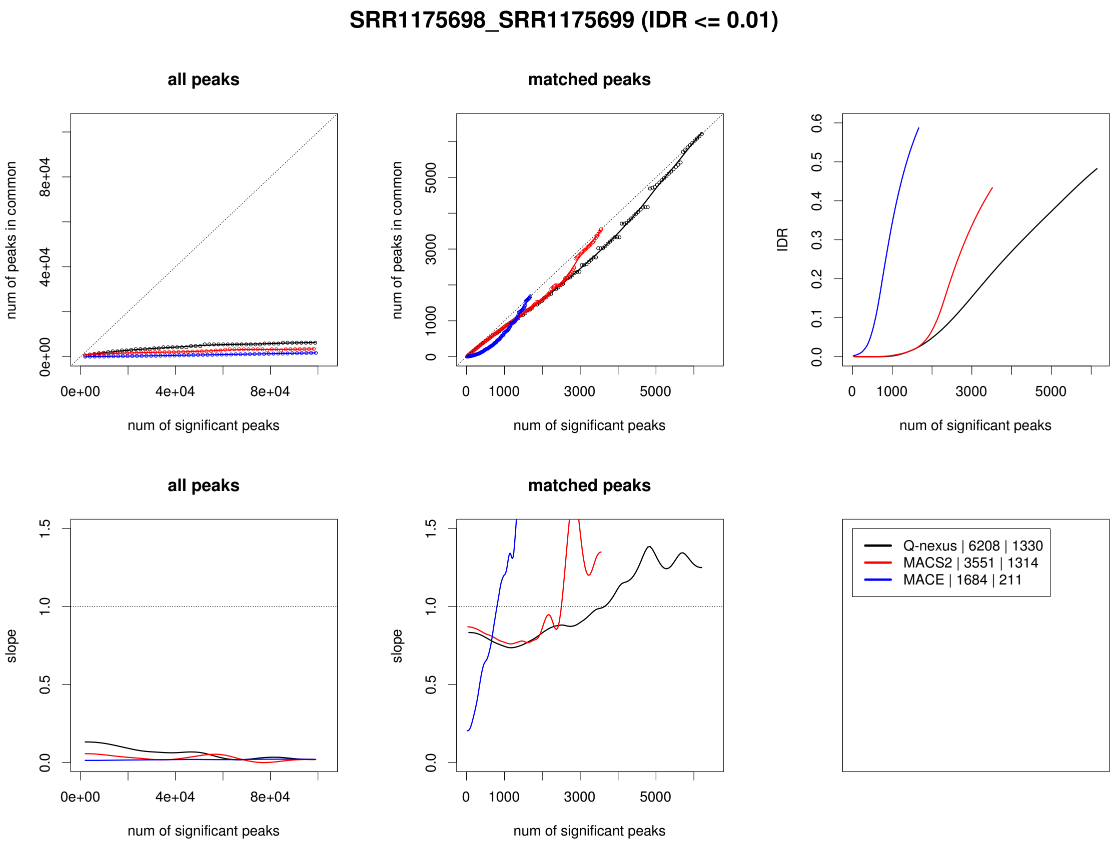

Figure S12. Correspondence and IDR - Dorsal. Results of the IDR analysis. See [1, 2] for a detailed explanation of the plots.

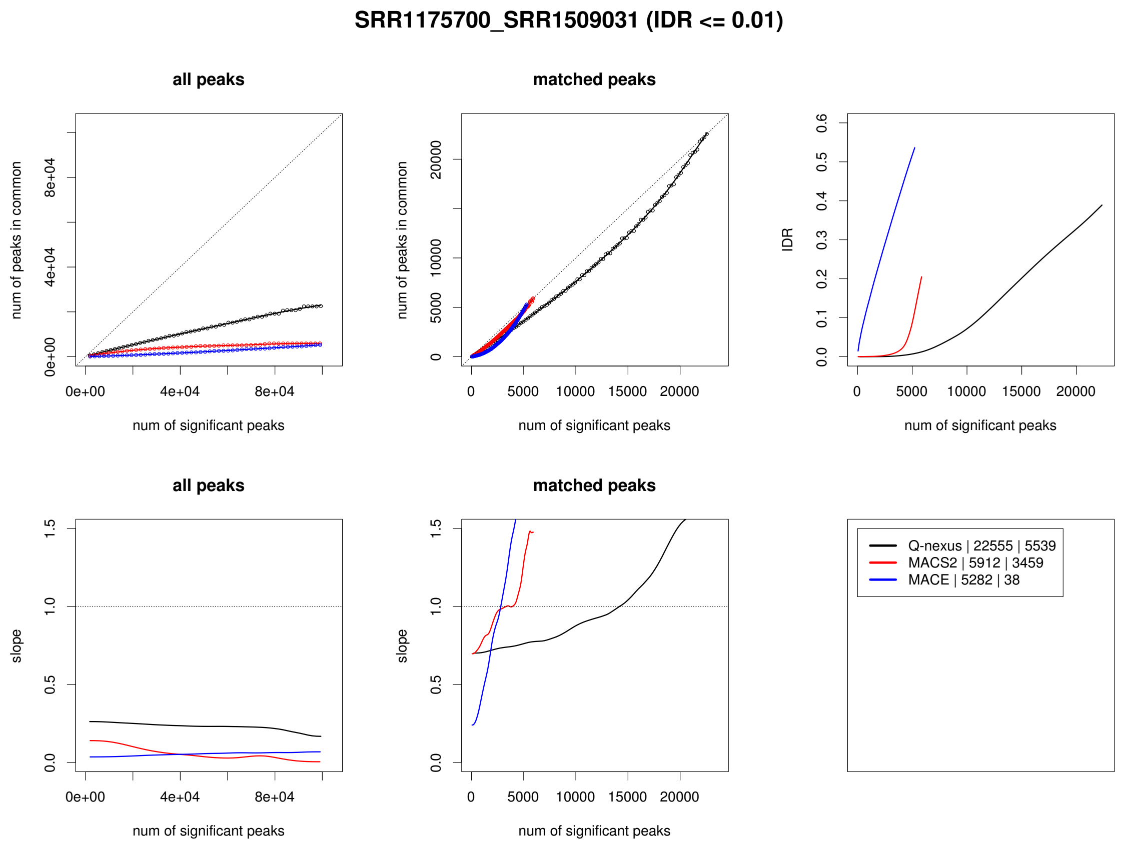

Figure S13. Correspondence and IDR - Max. Results of the IDR analysis. See [1, 2] for a detailed explanation of the plots.

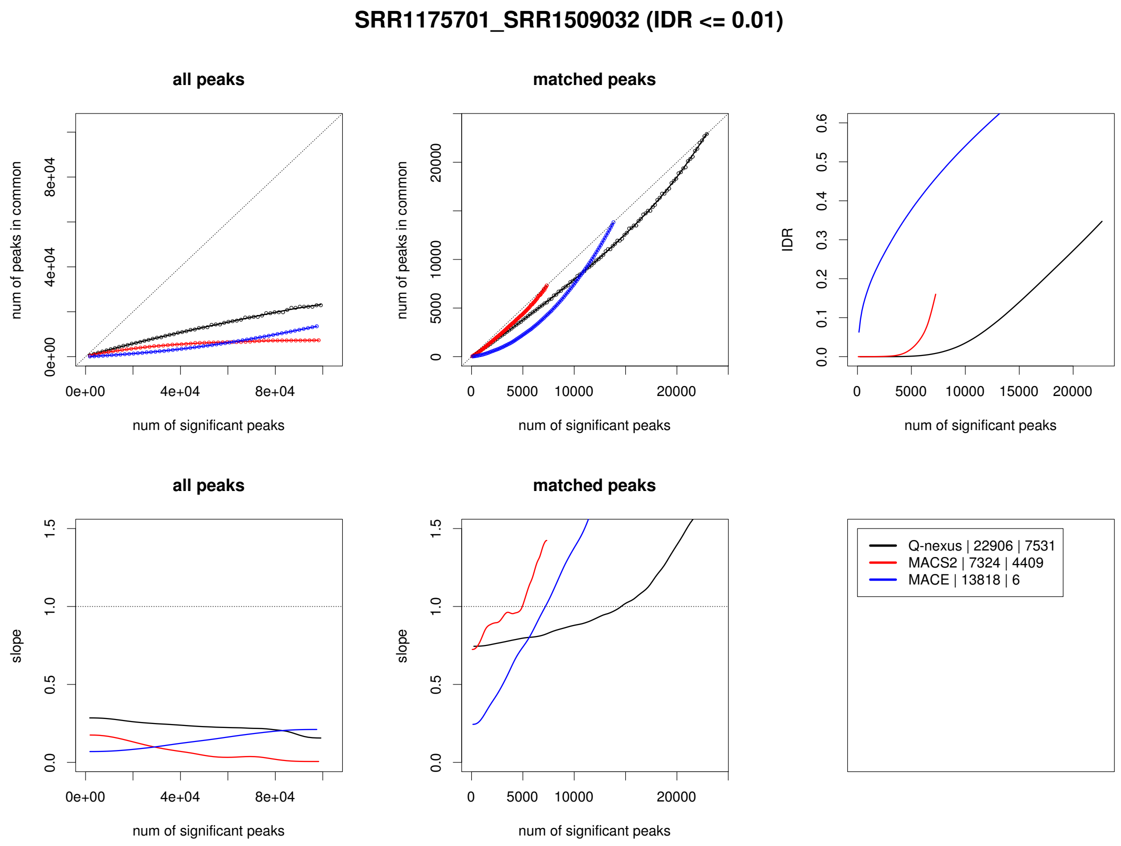

Figure S14. Correspondence and IDR - Myc. Results of the IDR analysis. See [1, 2] for a detailed explanation of the plots.

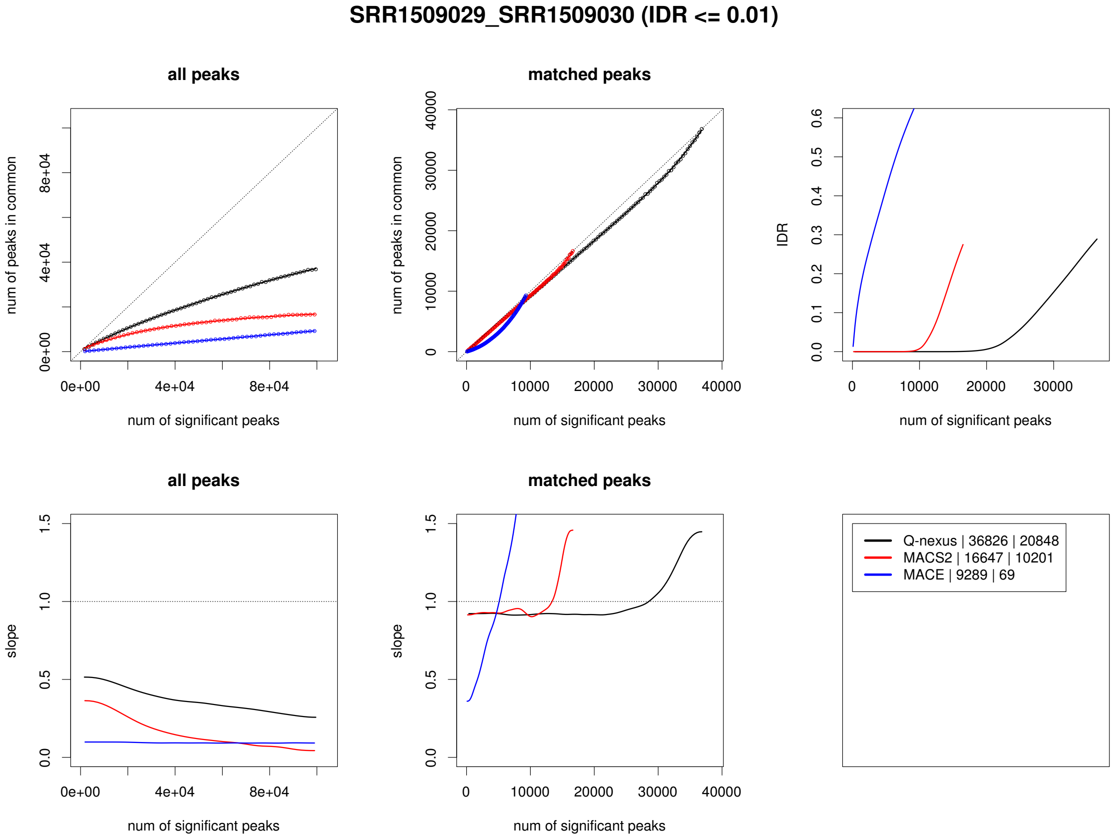

Figure S15. Correspondence and IDR - Twist. Results of the IDR analysis. See [1, 2] for a detailed explanation of the plots.

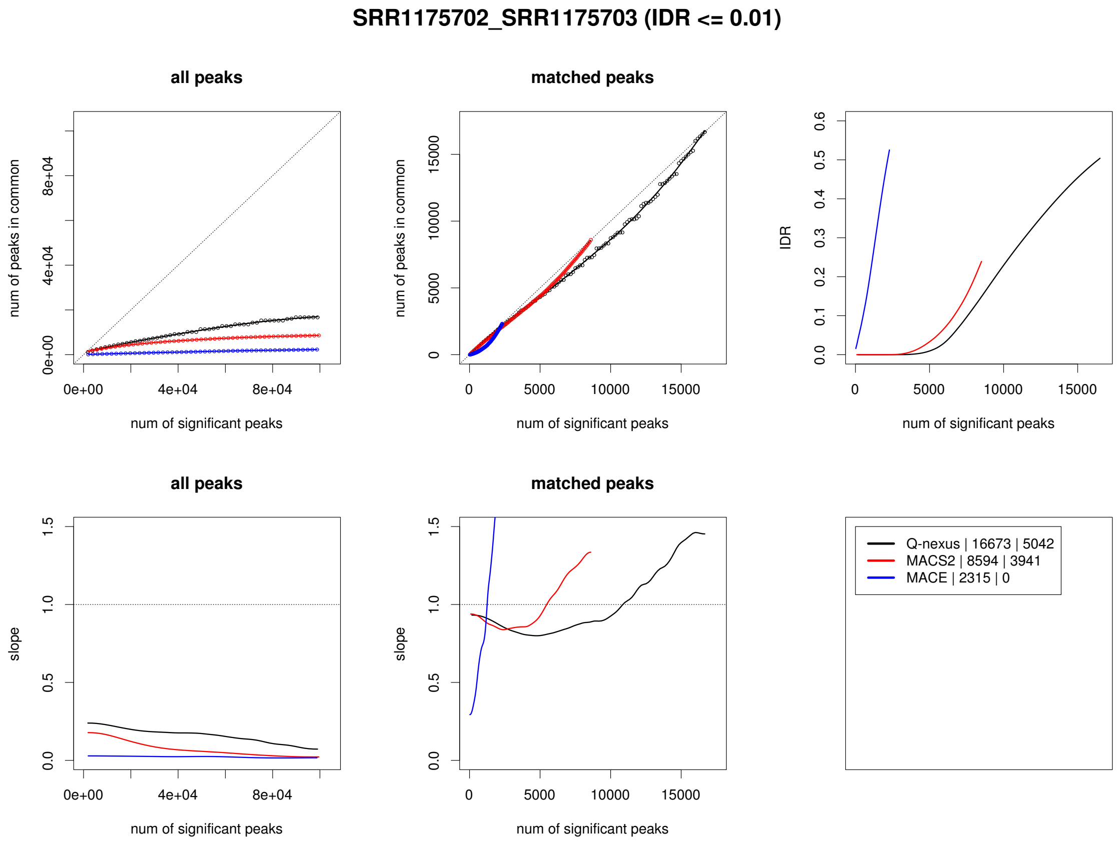

Figure S16. Correspondence and IDR - TBP. Results of the IDR analysis. See [1, 2] for a detailed explanation of the plots.

Table S1 Peak overlaps			
**Factor**	**Q-nexus**	**MACS2**	**MACE**
Dorsal	6,208	3,551	1,684
Max	22,555	5,912	5,282
Myc	22,906	7,324	13,818
Twist	36,826	16,647	9,289
TBP	16,673	8,594	2,315

Table S1. Peak overlap. The number of overlapping peaks among the 100,000 top ranked peaks of biological replicates.

Table S2 Peak overlaps with IDR $\leq$ 0.01			
**Factor**	**Q-nexus**	**MACS2**	**MACE**
Dorsal	1,330	1,314	211
Max	5,539	3,459	38
Myc	7,531	4,409	6
Twist	20,848	10,201	69
TBP	5,042	3,941	0

Table S2. Peak overlap. The number of peaks among the 100,000 top ranked peaks of biological replicates that overlap and have an IDR score ≤ 0.01.

References (Online Supplementary Material)

[1] Li, Q., Brown, B., Huang, H., Bickel, P.: IDR analysis 101 Measuring consistency between replicates in high-throughput experiments (2010)

[2] Hansen, P., Hecht, J., Ibrahim, D.M., Krannich, A., Truss, M., Robinson, P.N.: Saturation analysis of ChIP-seq data for reproducible identification of binding peaks. Genome Res **25**(9), 1391–1400 (2015). doi:10.1101/gr.189894.115
